# Acoustic, Phononic, Brillouin Light Scattering and Faraday Wave-Based Frequency Combs: Physical Foundations and Applications

**DOI:** 10.3390/s22103921

**Published:** 2022-05-22

**Authors:** Ivan S. Maksymov, Bui Quoc Huy Nguyen, Andrey Pototsky, Sergey Suslov

**Affiliations:** 1 Optical Sciences Centre, Swinburne University of Technology, Hawthorn, VIC 3122, Australia; 102831477@student.swin.edu.au; 2 Department of Mathematics, Swinburne University of Technology, Hawthorn, VIC 3122, Australia; apototskyy@swin.edu.au (A.P.); ssuslov@swin.edu.au (S.S.)

**Keywords:** acoustic frequency comb, phononic frequency comb, vibrations, nonlinear acoustics, acousto-optics, gas bubbles, liquid drops, Faraday waves, Brillouin light scattering, plasmonics, liquid metals

## Abstract

Frequency combs (FCs)—spectra containing equidistant coherent peaks—have enabled researchers and engineers to measure the frequencies of complex signals with high precision, thereby revolutionising the areas of sensing, metrology and communications and also benefiting the fundamental science. Although mostly optical FCs have found widespread applications thus far, in general FCs can be generated using waves other than light. Here, we review and summarise recent achievements in the emergent field of acoustic frequency combs (AFCs), including phononic FCs and relevant acousto-optical, Brillouin light scattering and Faraday wave-based techniques that have enabled the development of phonon lasers, quantum computers and advanced vibration sensors. In particular, our discussion is centred around potential applications of AFCs in precision measurements in various physical, chemical and biological systems in conditions where using light, and hence optical FCs, faces technical and fundamental limitations, which is, for example, the case in underwater distance measurements and biomedical imaging applications. This review article will also be of interest to readers seeking a discussion of specific theoretical aspects of different classes of AFCs. To that end, we support the mainstream discussion by the results of our original analysis and numerical simulations that can be used to design the spectra of AFCs generated using oscillations of gas bubbles in liquids, vibrations of liquid drops and plasmonic enhancement of Brillouin light scattering in metal nanostructures. We also discuss the application of non-toxic room-temperature liquid–metal alloys in the field of AFC generation.

## 1. Introduction and Motivation

Precision measurement underpins modern technologies that are critical for timing and communication as well as for fundamental science such as astrophysics. However, electronic and optical devices used in measurement systems generate interference (noise) that complicates or even prevents reading a pure signal. Thus, one of the main goals of precision measurement is to reduce the noise level by improving the signal-to-noise ratio and increasing the sensitivity of sensors and signal detectors.

Advances in optical technologies are essential for achieving these goals. Indeed, the unique physical properties of light and recent progress in the development of novel sources of light and synthesised optical materials enabling light manipulation at a nanoscale open unprecedented opportunities for reducing noise levels and increasing the measurement accuracy. For example, optical frequency combs (OFCs) (Section 2) have enabled scientists and engineers to measure and control light waves as if they were radio waves. Using OFCs, established technologies that employ radio and microwave frequencies—clocks, computers and telecommunications systems—can be seamlessly connected to devices that use optical waves with frequencies approximately 10,000 times higher than those of radio and microwaves [1,2,3].

**Figure 1:**
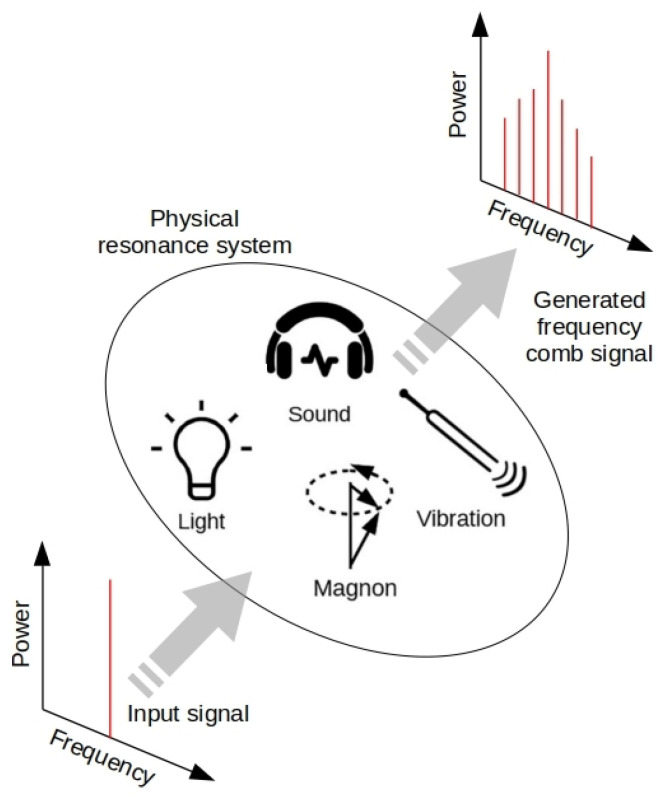
Conceptual illustration of the physical mechanisms of FC generation, where a resonant interaction of a single-frequency input signal with light, sound, vibrations and magnons—the quanta of spin waves—results in the appearance of equidistant coherent peaks in the power spectrum of the output signal. As demonstrated in this review article, an FC spectrum can be produced using either a sole resonant physical process or a combination of them, which is, for example, the case of Brillouin light scattering from phonons and magnons.

However, while optical technologies are an invaluable tool that researchers use to explore new horizons, they have a number of drawbacks that originate from fundamental physical limits. This is, for example, the case in underwater communication [4,5] and in some medical imaging and sensing modalities used deeply inside a living human body [6,7], where the intensity of light is dramatically attenuated due to scattering and optical absorption in liquids and bodily fluids and tissues. This situation has motivated pioneering studies of alternative approaches that use waves other than light to enable sensing and precision measurement in specific but critical areas of health studies and deep sea exploration. Given that optical waves share many fundamental physical properties with the waves of other nature—most notably with sound waves [8]—it has been suggested that certain optical precision measurement and sensing technologies, including OFCs, could be implemented using acoustic waves, vibrations and spin waves (Figure 1).

In this review article, we critically review results of recent studies, where novel acoustic frequency combs (AFCs)—non-optical counterparts of OFCs—have been introduced and a number of their potential applications suggested. We pursue several goals in the present review. Firstly, since the field of AFCs is relatively new, the terminology used in it is still not uniform and can vary from one paper to another (for example, although throughout the mainstream discussion we will use the term AFC, some authors prefer calling them phononic frequency combs [9]). Subsequently, one of our aims is to provide a taxonomy of AFCs and relevant concepts of non-optical frequency combs (FCs). Secondly, even though the seminal works on distinct non-optical FC technologies appeared almost simultaneously, they have resulted from rather isolated research efforts. While some of the AFC investigators have already consolidated their activities, which is, for example, the case in optomechanics and Brillouin light scattering communities [10,11] working on integrated photonic circuits [12,13], in general there is still no coherent research framework for the future development of AFCs. Therefore, the current review intends to promote collaboration between different research groups. Given this, the mainstream discussion of this article will specifically focus on the results that have received limited attention thus far but that, in our opinion, hold the promise to find their own application niche and influence further developments in the adjacent areas. Finally, to make the article accessible to non-specialists, the discussion of each specific AFC technique is accompanied by an overview of relevant physical phenomena with suggestions of further reading for researchers interested in more detail.

Thus, the remainder of this article is organised as follows. In Section 2, we discuss the origin of the concept of FCs and introduce the terminology used in this field of research [1,2,3]. In Section 3, we review electronically generated AFCs that have been employed in precision underwater measurements [5]. The recent advances in theory and application of phononic FCs [9,14,15] are discussed in detail in Section 4. In Section 5, we review FCs obtained using the Brillouin light scattering effect [16,17,18], and we also demonstrate the possibility of increasing the amplitude of spectral peaks in the comb spectra by means of plasmonic resonant effects supported by metal nanostructures [19,20]. The discussion of the novel approaches to the generation of AFCs continues in Section 6 and Section 7, where we review the recent advances in the fields of AFC generated using oscillations of gas bubbles in liquids [21,22] and vibrations of liquid drops [23,24], respectively. There, the reader will find a detailed analysis of both experimental and theoretical results [22] including those obtained using drops of room-temperature liquid metal alloys that have recently attracted significant attention in the fields of material science, electronics and optics [25,26,27,28]. The main discussion is concluded by an analysis of challenges faced by developers of the AFC technology and a discussion of the potential directions for future research. A list of abbreviations used in the main text is also given.

## 2. Optical Frequency Combs

An optical frequency comb (OFC) is a spectrum consisting of a series of discrete, equally spaced elements with a well-defined phase relationship between them. The foundations of this breakthrough technology were laid in the works led by the co-recipients of the 2005 Nobel Prize in Physics John Hall and Theodor Hänsch. Their contributions to the field of precision spectroscopy enabled measuring the light frequency with an unprecedented accuracy [29,30,31]. They and their collaborators demonstrated that a stable laser emitting light with a spectrum containing very fine colour (frequency) lines can be used in combination with an FC technique to measure the frequency of light very accurately. They also suggested that employing an FC technique enables measuring both time and distance more accurately than using any other approach.

In practice, the light frequency *f* that needs to be to determined may be too high to be measured directly. Subsequently, an indirect measurement technique was proposed, where one compares the unknown frequency to an optical ruler—an OFC [1,2,3]. The comparison between two light frequencies is made using a well-known beat technique that is based on measuring a frequency difference between the known and investigated waves, which is sufficiently small to be reliably measured using conventional methods.

**Figure 2:**
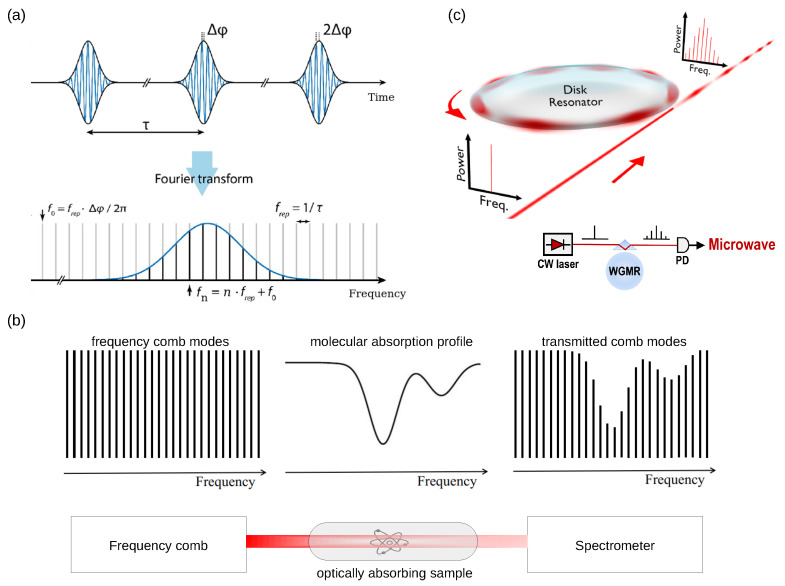
(**a**) Schematic of the OFC generation using a mode-locked laser. The top panel shows a train of optical pulses with a period $1/f_{rep}$. The bottom panel depicts the spectrum of narrow frequency peaks corresponding to the train of pulses in the time domain. The phase shift Δϕ of the carrier wave with respect to the pulse envelope induces a translation $f_{0}=f_{rep}\Delta \phi /2\pi$ of spectral peaks from their harmonic frequencies $nf_{rep}$. Reproduced from [2] with permission from Elsevier. (**b**) Sketch of an OFC-based spectroscopy technique. The OFC as a broadband light source interrogates an absorbing sample and a spectrometer analyses the transmission spectrum. (**c**) Artist’s rendition of a Kerr effect-based OFC generation in a micro-photonic disc resonator. A continuous-wave (CW) input pump wave creates a pattern of optical whispering-gallery modes (WGMs) inside the disc resonator, thereby inducing a periodic light intensity modulation and thus producing an output signal with an OFC-like spectrum. The bottom panel shows the sketch of a simplified experimental setup for the generation of a Kerr OFC and its further processing using a photodetector (PD). Reproduced from [33] published by De Gruyter Open under a Creative Commons License.

An OFC is typically generated using a mode-locked laser system [32], where, in the time domain (Figure 2a) [1,2], a train of ultrashort optical pulses is emitted. The period of the pulse envelope $1/f_{rep}=L/v_{g}$ corresponds to a round-trip time inside the laser cavity with a round-trip length *L* and the group speed of light $v_{g}$. Due to the dispersion of light inside the cavity, there is a phase shift Δϕ between the carrier and the pulse envelope signals. Hence, in the frequency domain (Figure 2a), the corresponding optical spectrum consists of a discrete set of equidistant narrow peaks with frequencies $f_{n}=nf_{rep}+f_{0}$, where *n* is a large integer indicating that the number of the peaks can be very high, $f_{rep}$ is the repetition frequency of the pulse envelope and $f_{0}$ is the carrier-envelope offset frequency that is related to the phase shift Δϕ as $f_{0}=f_{rep}\Delta \phi /2\pi$ [1,2].

Significantly, soon after the introduction of the concept of OFC, this technique found numerous applications beyond its originally intended use. For example, it was established that OFCs could provide long-term calibration of essential astronomical equipment [34], enable flexible control of ultrashort optical pulses [35] and benefit the generation of arbitrary radio-frequency waveforms and optical communications [36]. Of particular importance is also the application of OFCs in the field of spectroscopy [37,38], where, in an idealised OFC-based system (Figure 2b), an OFC both optically excites and interrogates the sample under study. The spectral response of a sample, which may arise due to linear or nonlinear absorption may span the entire OFC spectrum. Such a situation requires a spectrometer to conduct measurements. Therefore, existing spectrometers have been adapted and improved to resolve individual OFC peaks.

Alternatively, an OFC can be generated using a four-wave mixing (FWM) nonlinear-optical process [39], where, for example, a laser light at three frequencies $f_{1}$, $f_{2}$ and $f_{3}$ interacts in a nonlinear-optical medium, resulting in a new optical signal at a fourth frequency $f_{4}=f_{1}+f_{2}-f_{3}$. If the three original optical frequencies are a part of a perfectly spaced OFC spectrum, then the signal at the fourth frequency extends the already existing OFC spectrum. It is also possible to generate an OFC using laser light of two equally spaced frequencies, where FWM can generate light at different equally spaced frequencies via a cascaded nonlinear process. For example, such an interaction can produce light at a frequency $2f_{1}-f_{2}$ that, in turn, can subsequently participate in the nonlinear generation of additional new frequencies in the same OFC spectrum and so forth. This kind of cascaded OFC generation has been demonstrated in nonlinear optical fibres [40] and in some nanophotonic devices [41].

Other nonlinear optical processes such as second harmonic generation, where high-intensity pump light enters a nonlinear optical material and a weak optical signal is generated at a frequency twice that of the original pump light, or third harmonic generation of sum and difference frequency components, or intensity-dependent index of refraction (Kerr effect) [39] can be employed to create OFC [8]. In particular, the Kerr effect is used in an important class of Kerr OFCs or micro-combs [33,42,43], where a single laser is coupled with a photonic microresonator such as a glass disc that supports optical whispering-gallery modes. Although such resonant modes are not exactly equally spaced due to optical dispersion processes, they can be stabilised, for example, using the aforementioned FWM effect. Yet, it is noteworthy that, in the time domain, while mode-locked laser OFCs are virtually always associated with a series of short pulses, Kerr OFC exhibits complex phase relations between their individual modes that may not correspond to well-defined single pulses [44]. However, the modes of Kerr OFC remain highly coherent, thus enabling their application as pure OFCs.

For some practical applications, OFCs can be generated using an electro-optical modulation of a continuous wave laser light. Here, an OFC spectrum is obtained by modulating the amplitude or phase of a continuous wave laser source using an external modulator operating at a high radio or microwave frequency [45]. Given this, the spectrum of a so-generated OFC can be conveniently centred around an optical frequency of interest. Furthermore, this approach enables generating OFCs with higher repetition rates of more than 10 GHz, which is challenging to achieve using a mode-locked laser [36]. However, the number of peaks in the spectrum of electro-optical OFC is lower than in the spectrum of a mode-locked laser OFC.

Finally, we mention low radio-frequency OFCs generated using purely electronic and technically simpler devices that produce a series of pulses. Although such OFCs are used mostly in conjunction with some functions of electronic sampling oscilloscopes, they have been utilised in some optical domain applications, for example, in measurements involving laser diodes and in acoustic frequency combs proposed in [5] (Section 3).

## 3. Electronically Generated Acoustic Frequency Combs

Marine science has always been of technical, military and commercial importance. Since about 70% of the Earth’s surface is covered by water and the global mean sea level is rising due to climate change, exploration of oceans has become one of the priorities for both governmental and private sectors. In particular, in this area there is an urgent need for novel precision measurement techniques that would enable the exploration of deep sea, also facilitating the communication and data transfer between submarines and other equipment. However, in general, optical technologies cannot be used for these purposes because of a strong absorption of light in water. On the other hand, the attenuation of acoustic waves in water is much weaker. This property of sound makes it the prime candidate for use in underwater navigation and ranging (SONAR) and other applications.

In an idealised underwater distance measurement system, piezo-electric transducers driven by electric signals produce a train of acoustic signals and radiate them towards a target. If the speed of sound in water is known, the distance to a target is determined by measuring time between emitted and reflected acoustic pulses. However, the speed of sound in liquids depends on multiple variable environmental factors such as the ambient temperature and water salinity [4]. Therefore, its accurate value may not always be known. The acoustic measurement accuracy of an unlocked acoustic device relying on an incoherent data processing method is also intrinsically limited by destructive interference processes and a trade-off between spatial resolution and uncertainty in speed measurements, which prevents the current SONARs from resolving sub-centimetre distances. While such an inaccuracy is tolerated in some cases, in other applications (for example, in monitoring underwater glaciers) distance measurement precision of order of several millimetres is required [46].

**Figure 3:**
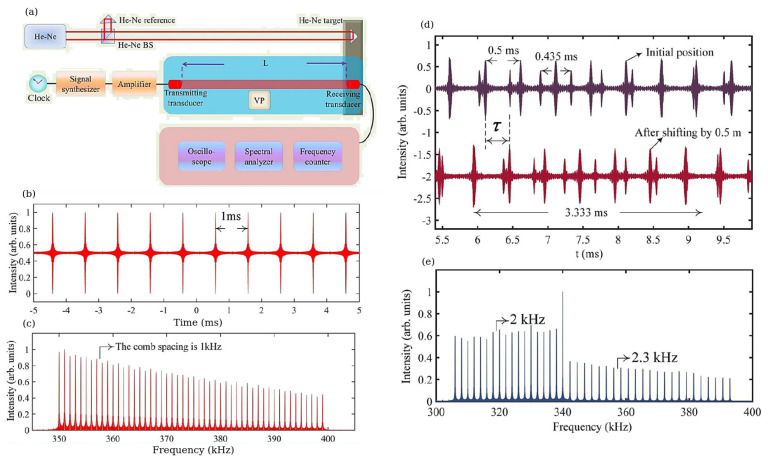
(**a**) Schematic of the experimental setup employed to generate an AFC using a Rb-clock-stabilised electronic FC and a set of piezo-electric transmitting transducers and subsequently exploit the resulting AFC for measuring the distance in an underwater environment (VP—sound velocity profiler; BS—beam splitter). (**b**) Acoustic pulse train in the time domain in the 350–399 kHz frequency range and (**c**) the AFC spectrum corresponding to it. (**d**) Waveforms detected by the receiving transducer in the time domain. The upper waveform was detected at the initial position and the lower one obtained after moving the receiving transducer by 0.5 m. (**e**) Spectrum of the waveform in panel (**d**) showing two different repetition frequencies. Reproduced from [5] with permission of John Wiley and Sons.

Subsequently, a novel approach to underwater distance measurement was proposed in [5], where (Figure 3a) a signal produced by a generator referenced to a Rb clock was first amplified and then used to drive a series of transmitting transducers with different nominal acoustic frequency bands. Receiving transducers were fixed at an a priori known distance, and its output signal was analysed using an oscilloscope, spectrum analyser and frequency counter. Since the response of piezo-electrical transducers closely follows the waveforms of the electric signals used to drive them, a comb-like spectrum could be generated using a driving signal that is an electronic FC itself (Section 2). A He-Ne laser was used as the reference interferometer in air—the He-Ne laser beam was aligned with the acoustic beam of the transducers. Significantly, since the peaks of the electrical FC spectrum are equally spaced and fully referenced, the corresponding acoustic frequencies emitted by the transducers and the repetition frequency are as stable as those of a Rb clock (Figure 3b,c).

The distance between the transmitting and receiving transducers was measured using a two-step protocol involving a coarse measurement followed by a refinement step. During the coarse measurement, the integer part of the pulse-to-pulse length of the AFC was determined using its repetition frequency $f_{rep}$. Subsequently, at the refinement step the fractional part of the pulse-to-pulse length was found using the slope of the unwrapped phase that is proportional to the time delay τ (the phase slope was measured using a Fourier transform-based approach). Finally, using these data the actual distance between the transducers was precisely determined.

The maximum unambiguous range of the discussed underwater measurement system is given by the longest range that a transmitted pulse can travel forward and back during the time between two consecutive transmitted pulses. The proposed method of AFC generation can be used to produce several AFCs at the same time. It has been demonstrated that this can be employed to expand the unambiguous measurement range. In particular, in [5], the transmitting transducers were set to simultaneously emit a pair of AFC signals with two different repetition frequencies, 2 and 2.3 kHz. To distinguish between the two AFCs, they had different amplitudes. Figure 3d,e shows the detected waveform of the receiving transducer, where the resulting dual-AFC signals can be seen as the pulse trains with a 0.5 ms period (2 kHz repetition frequency, 0.74 m pulse-to-pulse length) and a 0.435 ms period (2.3 kHz repetition frequency, 0.640 m pulse-to-pulse length). A signal with a larger period of 3.33 ms corresponds to the extended unambiguity range of 4.9 m. Using this measurement approach, underwater distance measurements up to 7 m with stable environmental conditions in an anechoic pool were conducted with a measurement uncertainty of approximately 50 μm compared with the optically measured reference values.

## 4. Phononic Frequency Combs

### 4.1. Micromechanical Resonator-Based Phononic FCs

In this section, we discuss another kind of AFC, phononic FCs [9,47,48,49,50,51,52], which exploit high-frequency nonlinear mechanical vibrations [53] to generate FC-like signals. The cited seminal works have been the first to demonstrate that the robustness and versatility of the Nobel Prize-winning OFC technology could be employed in the frequency ranges that are not accessible using light. Indeed, as shown in [1], despite a large number of research studies focused on the expansion of the spectral coverage of the existing OFC generators, there are no FC-like technologies that would operate in, for example, ultrasound (MHz) and hypersound (GHz) acoustic frequency ranges [54]. Although the hypersonic range remains insufficiently explored compared with audible sound and ultrasound, partly because GHz acoustic waves undergo a stronger attenuation [54], hypersound is of significant technological importance because in this frequency range one often observes intriguing physical effects such as Brillouin light scattering (BLS) which originates from a non-elastic light interaction with acoustic waves [55]. Similarly to Raman scattering [56], BLS underpins an emergent spectroscopy and imaging technique that has already found important applications in biology, medicine, chemistry, physics and material science [57,58,59,60]. We will return to this discussion in Section 5.

Although there are certain physical analogies between phonons and photons [8], in general a dispersion relation for phonons is nonlinear since it is related to acoustic and thermal properties of a material. As a result, the use of standard methods of OFC generation in phononic systems is frequently impossible. Therefore, novel approaches that are independent of the phonon dispersion relation are required. A solution to this problem was theoretically proposed in [47] using nonlinear phononic systems such as mechanical cantilevers [61] and chains of particles linked by springs that obey Hooke’s law but at the same time exhibit a nonlinear behaviour [62]. In such systems, several phonon modes can be simultaneously excited by an external driving force producing an FC-like spectrum with an array of discrete and equidistant spectral peaks corresponding to the frequencies of nonlinearly excited phonon modes. FCs generated using nonlinear resonance of different orders were theoretically investigated in [47] and a possibility of frequency stabilisation of the higher-order modes was demonstrated.

**Figure 4:**
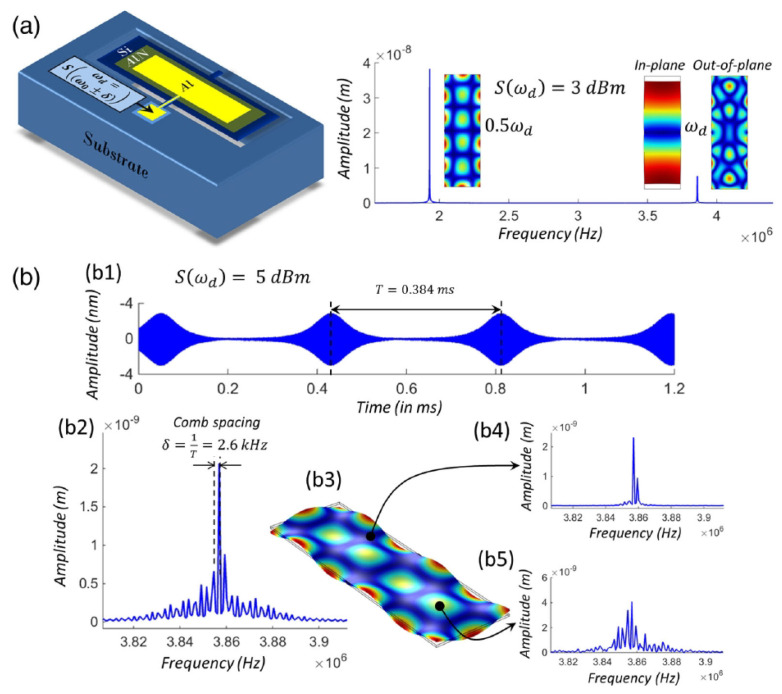
Generation of a phononic FC using nonlinear acoustic resonances in a piezo-electrically driven micromechanical resonator. (**a**) Left: Sketch of the micromechanical resonator. Right: Spectrum amplitude plot showing a parametric excitation of an out-of-plane subharmonic mode of the resonator that is tuned on its in-plane extensional mode using a driving signal $S(\omega_{d})$ = 3 dBm. Panels (**b1**,**b2**) show a pulse train corresponding to the FCs generated at $S(\omega_{d})$ = 5 dBm and its spectrum, respectively. One can see the peaks with an interpeak spacing of 2.6 kHz. Panel (**b3**) shows the displacement profile of the subharmonic mode. Panels (**b4**,**b5**) demonstrate that the FC generation is possible only using the displacement at an antinode of the subharmonic mode of the resonator. Reproduced with permission from [9]. Copyright 2017 by the American Physical Society.

Based on the theoretical results presented in [47], in the work [9], a phononic FC was experimentally created using a piezo-electrically driven micromechanical resonator, where an electromechanical coupling led to signal enhancement and, consequently, stronger nonlinearities, thereby reproducing the behaviour of nonlinearly oscillating particle chains considered in the model proposed in [47]. The micromechanical resonator was fabricated on a Si chip packaged in a ceramic leadless chip carrier (Figure 4a left). The resonator was driven by electrical signals produced by a waveform generator and its mechanical response (Figure 4b) was optically recorded by a laser Doppler vibrometer (LDV) [8]. At a high driving signal amplitude that exceeded a specific threshold value, the data obtained using the LDV and a spectrum analyser revealed the existence of an autoparametrically generated subharmonic mode (Figure 4a right). The analysis of the displacement profile of the resonator corresponding to that subharmonic mode (Figure 4b) demonstrated that tuning of the signal registration equipment on an antinode of the subharmonic mode would be advantageous for FC generation. Furthermore, according to the authors of [9], the result illustrated in Figure 4b speaks in favour of a phase coherency of equidistant FC peaks and thus their conceptual analogy with the peaks of Kerr OFCs. Since the FC generation was possible only when the amplitude of the driving signal exceeded a certain threshold value, further analysis was carried out revealing that when the driving amplitude was increased, the FC spectrum extended to higher orders. This important result shows that the spectral bandwidth of the so-generated FC is directly related to the driving amplitude level.

**Figure 5:**
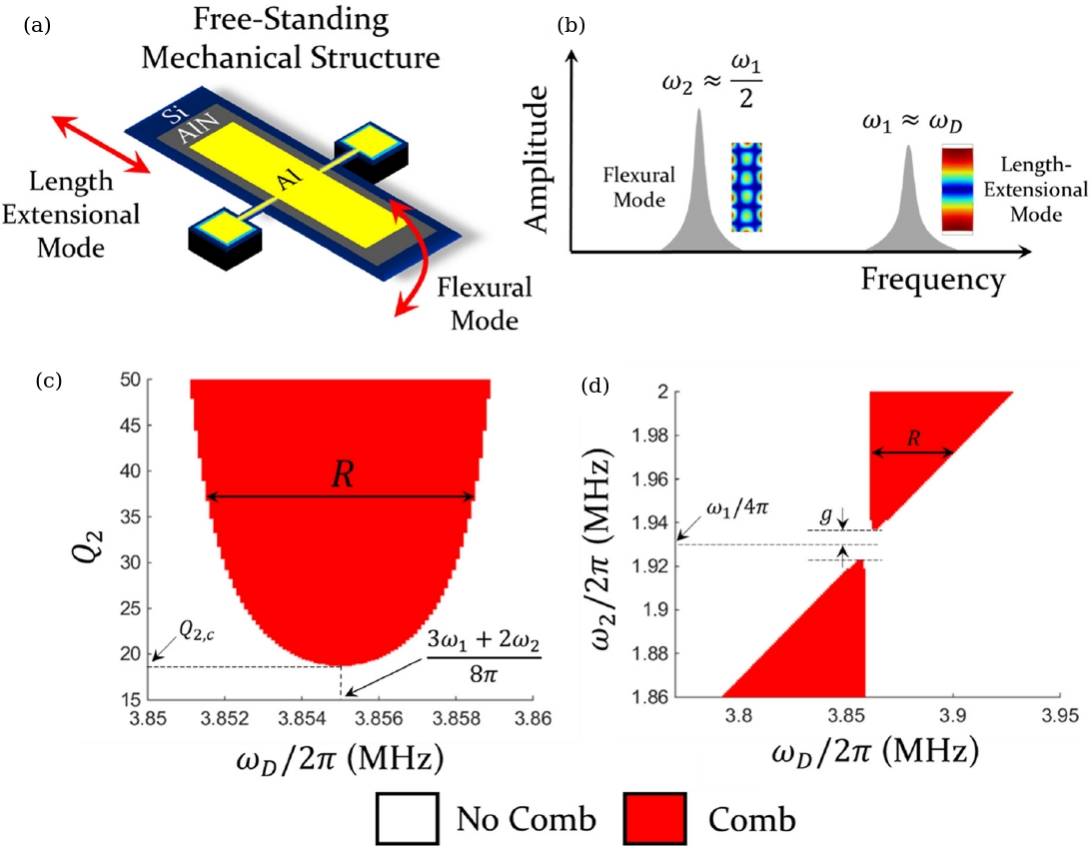
Model of phononic FC generation using nonlinear resonances of a micromechanical resonator. (**a**) Sketch of the model of the micromechanical resonator used in the experiment in [9]. (**b**) Schematic representation of the experimental spectrum in Figure 4a (right) showing a fundamental length-extensional mode of the resonator (the resonance frequency $\omega_{1}$) and its subharmonic flexular mode (the resonance frequency $\omega_{2}\approx \omega_{1}/2$.) (**c**,**d**). Graphical representation of the regions, where the generation of FC is possible using the experimental parameters from [9]. Panel (**c**) shows a plot of the quality factor of the mode with $\omega_{2}$ as a function of the driving frequency $\omega_{D}$. The $\omega_{2}$-vs-$\omega_{D}$ plot is presented in Panel (**d**). The parameters are $\omega_{1}/2\pi$ = 3.86 MHz and $Q_{1}$ = 4000 in (**c**) and $\omega_{1}/2\pi$ = 3.86 MHz, $Q_{1}$ = 4000 and $Q_{2}$ = 50 in (**d**). Reproduced from [14] with the permission of AIP Publishing.

In the follow-up work [14], the influence of the phonon mode structure on the FC generation was investigated using a model of two nonlinearly coupled phonon modes (Figure 5a,b). The model predicted the existence of a region within the amplitude-frequency space where the FC generation is possible (Figure 5c,d). The frequency range *R* corresponding to this region is given by the expression $R=\left|\omega_{2}-\frac{\omega_{1}}{2}\right|-\frac{\sqrt{2}\omega_{1}}{\sqrt{Q_{1}Q_{2}}}$, where $\omega_{1}$ is the resonance frequency of the fundamental length-extensional mode of the resonator, $\omega_{2}$ is the frequency of its subharmonic flexular mode and $Q_{1}$ and $Q_{2}$ are the respective quality factors (Figure 5a,b). One can see that $R\to \left|\omega_{2}-\frac{\omega_{1}}{2}\right|$ for large values of $Q_{2}$. There also exists a critical value for $\left|\omega_{2}-\frac{\omega_{1}}{2}\right|$ given by parameter $g=2\omega_{1}\sqrt{\frac{2}{Q_{1}Q_{2}}}$, which implies that for $\left|\omega_{2}-\frac{\omega_{1}}{2}\right|>g$ the frequency range of the FC existence *R* scales linearly with $\left|\omega_{2}-\frac{\omega_{1}}{2}\right|$ (Figure 5d).

It was found that the region of FC existence originates from a subset of the Arnold tongues [63], a phenomenon known in the context of the interaction between oscillators, where one oscillator drives another. In particular, Arnold tongues have been observed in a two-oscillator system, where one oscillator influences the other but not vice versa, which is typical of oscillators driven by a periodic force. Moreover, it was established that the spectral location and composition of the region of FC existence can be analytically defined in terms of resonance frequencies, quality factors and mode coupling strength of the mechanical resonator as well as by a detuning of the driving frequency from those of the mechanical resonances.

It is also noteworthy that the FCs discussed in this section have not been precisely stabilised to a frequency reference. Therefore, similarly to Kerr OFCs, they cannot be considered as counterparts of mode-locked laser OFCs (Section 2). This fact was perceived as a fundamental limitation for applications such as underwater distance measurements [5] because the modes of mechanically generated FCs could be incoherent. Nevertheless, the results discussed in this section have opened novel opportunities in the fields of ultrasensitive vibration detectors [64], phonon lasers [65,66], quantum computers [67] and imaging [8,9,47], where some incoherence of FCs can be inconsequential.

### 4.2. Phononic Frequency Combs in Bulk Acoustic Wave Systems

The discussion of mechanically generated FCs in Section 4.1 demonstrates that to achieve an FC-like signal in such systems one needs to design a certain mode structure and to apply a high threshold driving force. While these requirements can be fulfilled in many practical situations, their realisation may be impossible in some applications. For example, this is the case for a large group of technologies that include stabilised low-noise classical and atomic oscillators and measurement systems, high-sensitivity displacement sensors, high-precision electron spin and ferromagnetic resonance spectroscopy, high-precision measurement of material properties and high-quality-factor hybrid quantum systems [15,68,69]. The aforementioned technologies enable the realisation of precision measurement tools and techniques to test some of the core concepts of fundamental physics, such as modern searches for Lorentz invariance violations in the photonic [70], phononic [71,72] and gravity domains [73], variations in fundamental constants [74] and research on dark matter [75]. In these applications, bulk acoustic wave (BAW) devices have found very extensive applications. Moreover, both bulk and surface acoustic wave (SAW) devices have been used for spectroscopy, detection and sensing [76]. Nonlinear dynamics of BAW and SAW mechanical systems, including FC generation, has also become a subject of theoretical research [77].

**Figure 6:**
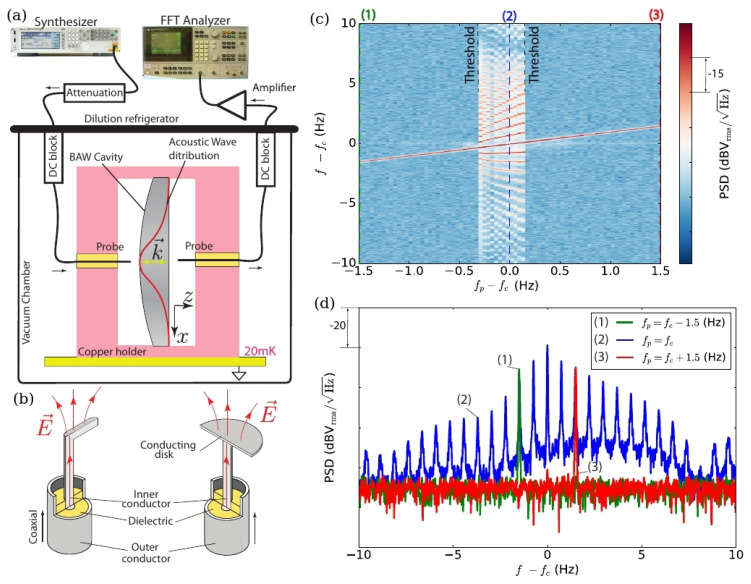
Generation of a BAW-based phononic FC. (**a**) Sketch of the experimental setup showing the BAW resonator, copper holder and excitation probes placed in a vacuum chamber. (**b**) Illustration of two types of excitation probes used in the experiment: L- (left) and disc-shaped (right) antennae. (**c**) A false-colour map composed of the individual output signal PSDs $S(f-f_{c})$ plotted as a function of the pump frequency detuning $f_{p}-f_{c}$ at a constant incident power P=−66 dB m. (**d**) Three PSD curves for different constant incident frequencies $f_{p}$ and the same power *P* corresponding to slices (1)–(3) along the vertical axis of the plot in panel (**d**). Reproduced from [15] published by the American Physical Society under the terms of the Creative Commons Attribution 4.0 International license.

Recently, the generation of phononic FCs in a BAW system at a temperature of 20 mK using a single-frequency low-power signal source was demonstrated [15]. To enable such a generation, in general one needs a system with low losses and strong nonlinear effects. To achieve this, a phonon-trapping stress compensated quartz BAW cavity operating at 20 mK was employed (Figure 6a). Quartz BAW cavities are known to have extremely high values of quality factors at cryogenic temperatures reaching $Q=8\times 10^{9}$ [78]. At the same time, quartz BAW cavities possess significant acousto-mechanical nonlinearities that originate from the lattice non-harmonicity [79] that can be described using a Duffing oscillator—a nonlinear second-order differential equation used to model certain damped and driven oscillations [63]. There are also other sources of nonlinearity originating from thermoelectroelastic effects and physical processes, which are observed mostly at milli-Kelvin temperatures, such as coupling with ensembles of two level systems (TLSs) [80]. In particular, the presence of TLSs has been demonstrated in BAW cavities through a number of effects including nonlinear losses and magnetic hysteresis. However, these processes hinder the generation of FCs and should be avoided.

This approach was adopted in the experiment reported in [15], where the BAW cavity was placed inside a copper holder attached to a base plate of a refrigerator cooled to 20 mK. To ensure that no coupling exists between the holder and BAW the fundamental microwave resonance frequency of the former was chosen to be much higher than any resonance frequency of BAW. The acoustic modes were excited and detected piezo-electrically using two specially designed coaxial probes coupled to the electric field of the structure (Figure 6b).

In one of the experiments reported in [15], the system response was analysed in terms of the signal power spectral density (PSD) S(f) measured as a function of the pump signal frequency $f_{p}$ in the vicinity of the acoustic resonance $f_{s}$ at a constant signal power *P*, see Figure 6c, where a false-colour map is composed of individual PSD curves of the output signal for each incident signal frequency $f_{p}$ used in the experiment. Note that each PSD curve was obtained independently with a time delay between two consecutive measurements sufficient to suppress any residual signals coming from a preceding measurement. It is also noteworthy that the experimental setup was stable on the time scale of the measurements since it was locked to an atomic frequency standard, and that the characteristics of the BAW resonator were found to be insensitive to possible temperature fluctuations during the measurement.

Figure 6d shows three PSD curves obtained for different values of the incident signal frequency $f_{p}$ at a constant power *P* corresponding to slices (1)–(3) along the vertical axis in Figure 6c. An FC is generated when the pump signal frequency approaches the resonance frequency. Furthermore, the FC exhibits two thresholds on each side of the resonance and the FC repetition rate is about 0.8 Hz when $f_{p}=f_{c}$. In the subsequent experiments reported in [15] the same threshold of the FC generation was observed when the incident power was varied but the excitation frequency was $f_{p}\approx f_{c}$. The analysis of the experimental results also revealed that the FC spectrum significantly depends on geometry of excitation and detection electrodes, Figure 6b. Yet, the fact that a strong Duffing nonlinearity was observed below the generation threshold indicates that the system is a phononic analogue to Kerr OFCs excited in monolithic optical microresonators (Section 2). Thus, it was concluded that the ultralow power regime explored in [15] opens a way for integrating a BAW-based phononic system with a quantum hybrid counterpart such as superconducting qubits.

## 5. Brillouin Light Scattering-Based Frequency Combs

In this section, we discuss the recent achievements in the developing field of FC generation using Brillouin light scattering (BLS), a physical effect named after Léon Brillouin, where light interacts with material waves in a medium [54]. Such an interaction is enabled by a dependence of the optical refractive index on the material properties of the medium. For example, it is well established that the refraction index of a transparent material changes when it is mechanically deformed. As a result of a deformation, a small fraction of light that is transmitted through the material or reflected from it changes its momentum (i.e., its frequency and energy are changed). This process is similar to an effect where diffraction of light is caused by diffraction grating, the components of which vibrate with a frequency that is much smaller than the frequency of the light wave. In solid media, macromolecular aggregates, biological media and liquids and gases, BLS can be observed as a result of light interaction with acoustic (phononic) modes [35,54,56,58,59], exciton-polariton (a hybrid light and matter quasiparticle arising from a strong coupling of the electromagnetic dipolar oscillations and photon) [81] and spin waves and their quanta—magnons [82]—existing in magnetic materials [83,84,85,86,87,88].

Although Rayleigh scattering can also be considered to be due to fluctuations in the density of an optical medium, thus leading to variations in its refraction index, such fluctuations are of random and incoherent nature. In contrast, BLS is caused by correlated periodic fluctuations such as phonons and magnons. Therefore, Rayleigh scattering involves no energy loss or gain. On the other hand, although Raman scattering also involves inelastic interaction processes caused by vibrational properties of matter, the range of frequency shifts associated with this effect are very different compared with those in BLS. Thus, BLS and Raman scattering provide very different information about the sample under study: Raman spectroscopy enables one to determine the chemical composition and molecular structure of the medium while BLS senses the elastic properties of the material [58,89].

**Figure 7:**
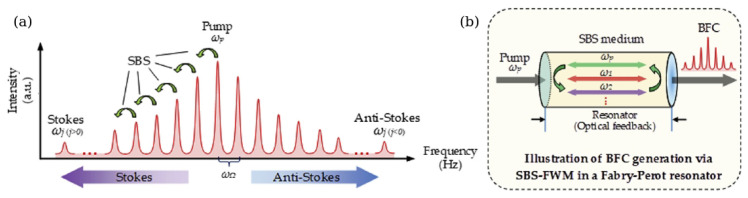
Illustration of the generation of an SBS-based FC. (**a**) The spectrum of an SBS FC showing the Stokes and anti-Stokes frequency peaks generated due to the SBS effect. (**b**) Illustration of a cascaded SBS effect combined with FWM arising from the optical Kerr-nonlinearity. Reproduced from [17] with permission of Elsevier.

Before we discuss FCs generated using BLS, we also note a conceptual difference between this effect and stimulated Brillouin scattering (SBS) [17,55,90]. SBS arises when an intense beam of laser light propagates through an optical medium, such as an optical fibre, and when variations in the optical electric field of the beam itself induce acoustic vibrations in the medium via electrostriction and radiation pressure effects. Under these conditions, the beam may display BLS as a result of the interaction with the vibrations, leading to the generation of optical signals with a spectrum consisting of a large number of equally spaced peaks that are coherently phased—a Brillouin OFC (BFC) (Figure 7) [10,17,91,92,93,94]. However, the so-generated FCs belong to the group of OFCs since SBS is mostly a nonlinear-optical effect that was discovered only after the invention of a laser [17,39,55]. Therefore, we refer an interested reader to the cited studies and references therein while in the following we focus on FCs based on the original BLS effect.

### 5.1. Magnonic BLS-Based Frequency Combs

While BLS offers significant advantages in several research fields, in general this technique requires sophisticated experimental instrumentation to reliably detect light scattering frequency shifts. A typical frequency shift observed in BLS measurements ranges from several MHz to several GHz, which is very small compared to the frequency of the incident light (several hundreds of THz). As a result, in an optical spectrum, the BLS peaks are located on the shoulders of the central Rayleigh scattering peak and their amplitudes are so small that to resolve them a Sandercock multi-pass Fabry–Pérot interferometer [95] or a virtual-image phase array (VIPA) spectrometer has to be used [96]. However, despite these technical challenges, BLS spectroscopy has been an essential tool for research on spin wave excitation in ferromagnetic micro- and nano-structures [83,84,85,86,87,88] and on phonon excitations in solid and biological media [35,54,56,58,59,89].

**Figure 8:**
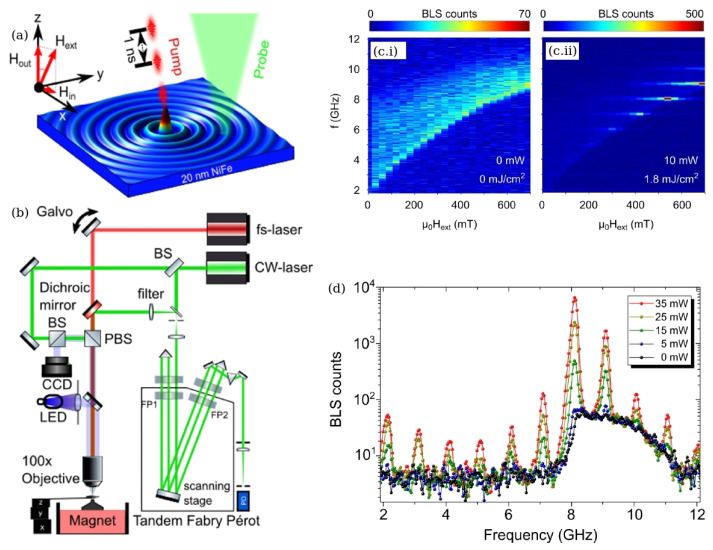
Illustration of the generation of a magnonic BLS-based FC. (**a**) A ferromagnetic thin film sample is pumped with a red (816 nm wavelength) 120 fs-long pulsed laser light and probed using a continuous green laser (532 nm) light. The direction of the external magnetic field $H_{ext}$ applied to the sample is indicated. (**b**) Schematic of the optical setup of the pump-probe experiment, where BS denotes a 50/50 beam splitter and PBS is a polarising beam splitter. The sample is placed right below a microscope objective to achieve diffraction-limited focusing. The backscattered light is analysed using a six-pass tandem Fabry–Pérot interferometer (TFPI) and detected using a single-channel avalanche photodiode (APD). False-colour maps of thermally excited spin wave spectra as a function of the applied magnetic field without (**c.i**) and with (**c.ii**) irradiation with an fs-laser pulse train at a 1.8 mJ/cm^2^ fluence. (**d**) BLS counts as a function of frequency at an applied magnetic field magnitude of 600 mT for four different laser powers. The thermal spin wave background is also shown. Reproduced from [18] published by the American Physical Society under the terms of the Creative Commons Attribution 4.0 International license.

Recently, the BLS spectroscopy has been employed to produce FC-like signals originating from spin wave modes excited in a ferromagnetic thin film structure [18,97]. In those works, a thin Permalloy (${\mathrm{Ni}}_{80}{\mathrm{Fe}}_{20}$ alloy) film—a standard building block of many magnonic and spintronic devices [82]—was deposited onto a sapphire substrate using a DC magnetron sputtering technique. A sapphire substrate was chosen due to its negligibly small optical absorption at the frequency of the laser light used in the BLS setup and also due to its high thermal conductivity. The fabricated films were characterised using a pump-probe experimental setup schematically shown in Figure 8a,b, where the pump beam was emitted by a mode-locked laser with a 1 GHz repetition rate at a wavelength of 816 nm with a 30 fs pulse duration and pulse energies of up to 1 nJ. Since the emitted laser pulse stretches during the propagation in the optical system, its actual duration at the moment of time when it reached the sample was 120 fs. A microscope objective was used to focus the laser beam into a spot with the size approaching the optical diffraction limit (approximately 400 nm). The magnetisation dynamics in a Permalloy film was probed using a single-frequency 532 nm laser light that was also focused to the diffraction limit using the same microscope objective. The probe light scattered backwards from the sample was first collected and filtered using a polariser and then analysed using a six-pass tandem Sandercock Fabry–Pérot interferometer and then detected using a single channel avalanche photodiode. The pump laser beam was scanned over the sample using a pair of galvanometer mirrors and lenses that enabled changing the lateral distance between the pump and the probe beams.

Figure 8c.i shows the typical field dependence of the thermal spin wave spectrum obtained for a thin Permalloy film sample when the pump laser was turned off. In this figure, a sharp cutoff of the spin wave band is seen that is in good agreement with the frequency-vs-applied magnetic field dependence theoretically predicted by the Kittel equation [88]. The corresponding spin wave spectrum at the same location of the Permalloy film but with the pump laser turned on is shown in Figure 8c.ii. In this case, the spectrum dramatically changes its character by gaining a number of distinct equally spaced peaks that appear with the 1 GHz repetition rate. More detailed information about the generated FC-line spectrum is given in Figure 8d, which shows the BLS counts as a function of the frequency at an applied magnetic field of 600 mT for four different laser powers, also showing the thermal spin wave background. From this result, one can deduce that 5 mW is the threshold power level for the FC generation.

Note that studies reported in [18,97] did not aim to generate FC signals for use in applications where other kinds FCs have been typically used. Instead, a method of FC-enhanced BLS microscopy was introduced to coherently excite vibrational and spin wave modes in the sample. This new approach is more advanced than a conventional impulse-driven stimulated BLS, where the spatial resolution is limited by the size of a virtual grating induced in the medium by the pump laser beam. Nevertheless, those results are of immediate relevance to the mainstream discussion in the current review article since they facilitate the development of BLS-based FC techniques and promote a deeper understanding of fundamental physical processes that underpin their operation. These include the enhancement of weak BLS signals using surface plasmon resonances supported by metal thin films, gratings and nanostructures [7,68,88] that we discuss in subsequent sections.

### 5.2. Plasmon-Enhanced Brillouin Light Scattering Effect

Surface plasmons are optical waves that propagate along a metal–dielectric interface [98,99]. A localised surface plasmon mode is a special case of a surface plasmon wave that is confined to a metal grating or a nanoparticle. Typically, to create conditions favourable for the excitation of localised plasmons, at least one dimension of the structure supporting them must be comparable with or smaller than the wavelength of the incident light. For example, localised plasmons have been observed in spherical nanoparticles with a diameter of 10–50 nm and in nanorods that are 50–200 nm long and typically have a diameter of about 20 nm. The optical properties of localised plasmons have been used to enable many essential operations of light control and manipulations at the nanoscale. For example, localised plasmons have been exploited to dramatically enhance the local optical electric field and then use it to enhance the light–matter interaction processes that are essential for achieving strong nonlinear effects [100,101] and high sensitivity [102] in many practical situations.

Usually, nanoparticles and nanostructures supporting plasmon modes are made of gold or silver because these two metals exhibit relatively low absorption losses at the optical frequencies. Nevertheless, in many practical situations, gold and silver are combined with or substituted by ferromagnetic metals such as nickel, cobalt, iron and their alloys (e.g., Permaloy [103]). Although the absorption losses in plasmon structures made of these materials can be even higher than in devices made of pure gold or silver, the ferromagnetic metals and their alloys exhibit significant magneto-optical activity [104], which opens up avenues for ultra-fast control of light and high-sensitivity biosensing, thus laying a foundation of the field of magneto-plasmonics [7,88,105,106,107,108,109,110].

Advances in magneto-plasmonics are also relevant to the current discussion of BLS-generated FCs. Indeed, in a typical magnonic BLS experiment, the dispersion relationship of spin waves is determined by using *p*-polarised incident monochromatic light illuminating the sample at an angle θ that is linked to the wave vector $k_{SW}$ of the probed spin wave via the relationship $k_{SW}=\left(4\pi /\lambda \right)\sin\theta$, where λ is the wavelength of the incident light. Note that the same polarisation of the incident light is required for the excitation of surface plasmons in thin films and grating [98], including those made of Permalloy [88,103] (such grating is called magnonic crystals in magnonics). Subsequently, by analogy with the plasmon-enhanced magneto-optical response, the conditions for a resonant enhancement of the BLS signal due to surface plasmons can be satisfied in BLS measurements of ferromagnetic metal structures.

While this approach has not yet been validated experimentally, experimental evidence speaks for its plausibility. In fact, in magnonic BLS experiments, the interaction of light with magnetic modes in the sample is mediated by the magneto-optical effects such as the magneto-optical Kerr effect (MOKE) and the Faraday effect [83,104,107,109,110]. Here, MOKE represents a change in the polarisation and intensity of light that is reflected from the surface of a magnetised material. Similar to the Faraday effect, MOKE originates from the off-diagonal dielectric permittivity tensor components of the investigated magnetised material [104]. However, while the Faraday effect is observed in transmission and, consequently, occurs only in optically transparent materials, the observation of MOKE is possible mostly in highly optically reflecting samples. Given this, MOKE has been found to be especially suitable for studying magnetism of highly reflective metals. Since plasmon modes can be enhanced in the same metal structures, where MOKE is observed, it has been shown that the amplitude of the MOKE signal can be increased using both surface and localised plasmon waves [88,103,105,107,108,109,110]. Thus, since MOKE is also the main physical mechanism contributing to BLS and since its strength can be increased using a magneto-plasmonic technique, it is plausible that the BLS signal would also be amplified by a plasmonic interaction.

Another significant argument in favour of this assumption is an experimental demonstration of a plasmon-enhanced phononic BLS [16]. While phononic BLS is conceptually similar to the magnonic one, physical processes that underpin it are simpler compared with the complex magneto-optical effects, which facilitates magnonic BLS. From the experimental point of view, realisation of a BLS experiment involving a measurement of plasmonic enhancement is technically simpler in phononic BLS than in the magnonic one since a magnetic field needed to magnetise a sample in the latter case is not required (such a field is often created by bulky and expensive electromagnets that can also obstruct the sample from the source of light and photodetectors receiving it [103]).

**Figure 9:**
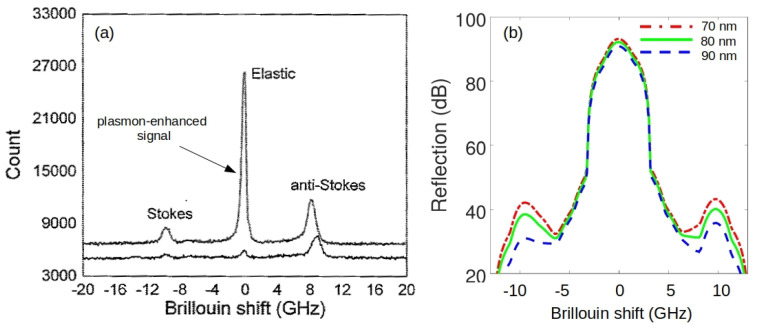
(**a**) Typical experimental BLS spectra with and without a gold disc nanostructure obtained after 5 s integration time using the excitation laser power of 40 mW. Note a significant signal enhancement due to the plasmon modes supported by the disc nanostructure. Reproduced from [16] with permission of SPIE and the corresponding author of this publication. (**b**) BLS spectra calculated using a 3D finite-difference time-domain model of the plasmon BLS effect for different disc diameters. Due to high computational demands, the integration time in the model was limited to 0.5 ms and window filtering was used, which increased the linewidth of the peaks. Note that the model reproduces the fact that in the experiment the amplitude of the anti-Stokes peak is higher than that of the Stokes peak. The BLS signal without the nanodiscs was not calculated.

A novel approach resolving the issue of a low strength BLS signal and enabling one to overcome such drawbacks of BLS spectroscopy as a long acquisition time and poor spectral resolution was proposed in [16]. There, the enhancement of BLS at the light wavelength of 532 nm was investigated using various acoustic modes of an alkaline-earth boroaluminosilicate glass plate coated with periodic arrays of gold nanodiscs that support localised surface plasmon modes. A similar enhancement was also observed from the bulk phonons, when the gold nanodiscs were covered by liquids such as methanol and water. The observed enhancement (Figure 9a) was attributed to the excitation of a fundamental plasmon mode of the array of the nanodisc, which was confirmed by the fact that no enhancement was observed without the nanostructure and that the enhancement of BLS was a function of the nanodisc aspect ratio and diameter. It was suggested that the demonstrated plasmonic enhancement could be combined with the virtually imaged phased array (VIPA)-based background-free BLS spectroscopy to optimise the acquisition time, and that an array of nanodiscs could serve as a platform for a practical implementation of the surface-enhanced BLS technique analogous to the well-established surface-enhanced Raman spectroscopy (SERS) [56,111,112].

To further investigate the origin of the plasmon-enhanced BLS process, we numerically modelled the BLS interaction in nanodiscs covered by water using a finite-difference time-domain method [19]. Figure 9b shows the calculated BLS spectra for three different disc diameters that were used in experiments reported in [16]. The model reproduces a plasmonic enhancement of the BLS signal and demonstrates that the amplitude of Stokes and anti-Stokes peaks depends on the nanodisc geometry. Note that the BLS signal was not modelled without a nanodisc structure and that in the model the integration time (0.5 ms) was much smaller than in the experiment (5 s) due to computational constraints. Nevertheless, the model was able to reproduce the experimental fact that the amplitude of the anti-Stokes peak is higher than that of the Stokes peak. This speaks in favour of its physical veracity. Note also that the linewidth of the peaks in the spectra in Figure 9b is artificially broadened due to the use of a window filter while postprocessing the simulated BLS signals. However, even though this artefact has complicated the analysis of the impact of the plasmon enhancement on the peak linewidth, a close inspection of the peaks reveals the signs of a dependence of both linewidth and Brillouin shift on the nanodisc geometry, which was also observed experimentally [16].

**Figure 10:**
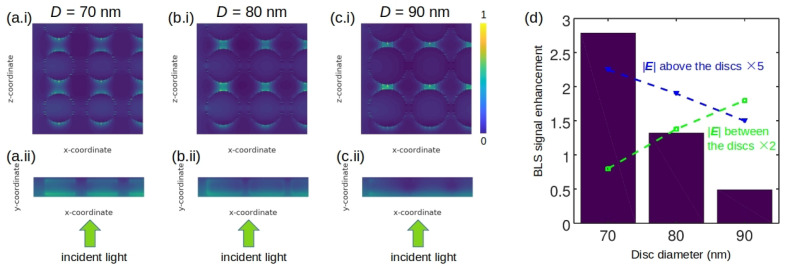
(**a–c**) Top and side views of the simulated optical electric field amplitude |**E**| of the plasmonic modes in the nanodisc array with different disc radii *D*. The direction of propagation of the 532 nm-wavelength incident light is indicated. (**d**) Simulated BLS enhancement as a function of the disc diameter *D*. The triangles and squares and the straight lines (guides to the eye) corresponding to them show the dependence of the field enhancement above and between the discs, respectively.

To gain a further insight into the origin of the plasmon-enhanced BLS, we simulated the scenario of a 532 nm wavelength light that is normally incident on a 5 × 5 nanodisc array covered by water and that is polarised along the *z*-axis of the coordinate system adopted in Figure 10a–c. In this model, the symmetry of the disc array was used to reduce computational effort. The spacing between the disc centres was fixed at 100 nm while their diameter *D* was varied. Figure 10a–c show that the enlargement of the disk diameters slightly enhances the amplitude of the local optical electric field |**E**| in the gaps between them. However, this effect plays an adverse role in achieving a stronger BLS response since the light localised in the gaps also penetrates the metal surface of the discs, which results in higher absorption losses. To demonstrate this, in Figure 10d we use bars to represent the BLS enhancement obtained from the anti-Stokes peak of the simulated spectra. The blue line connecting triangles shows the dependence of the field enhancement on the disc diameter calculated immediately above the discs, i.e., in the areas where light senses the refractive index modulation induced by phonons in the water layer. The green line connecting squares depicts the field enhancement in the gaps between the discs. We conclude that plasmonic enhancement of the BLS signal is possible when light is localised above the discs and not in the gaps between them. Conversely, the BLS signal is reduced when light is localised between the discs. This effect is especially pronounced in the case of large disc diameters D=90 nm and very small 10-nm-wide gaps between the discs, where our simulations predict a sharp decrease in the amplitude of the BLS signal compared with the predictions made for the arrays of nanodiscs with smaller diameters.

### 5.3. Application of Plasmon-Enhanced BLS in Frequency Comb Generation

As follows from the discussion in the previous section, the nanodisc geometry is not optimal for enhancing the BLS signal. Therefore, further analysis was performed in [19], where it was shown that a stronger plasmonic enhancement of the BLS effect could be achieved using elongated metal nanostructures such as plasmonic nanorods made of gold or silver. Similar to the well-known Fabry–Pérot resonators, long nanorods can support higher-order plasmonic modes (those modes do not exist in short nanorods that support only a dipole-like fundamental mode similar to the fundamental mode of nanodiscs). The operation based on a higher-order mode is expected to be advantageous for enhancing BLS signals since such modes reflect from a nanorod end multiple times. This effectively increases the interaction time of light with an acoustic wave that propagates in the bulk of surrounding water. Furthermore, the tight confinement of the optical electric field to the metal surface of the nanorod gives rise to an increased sensitivity of the plasmon resonance to changes in the dielectric permittivity caused by the propagation of acoustic waves occurring in close proximity of the nanorod [19]. At the same time, the light localisation associated with the excitation of the higher-order modes results in smaller absorption losses compared with the light confinement in small gaps between the nanodiscs (Figure 10) since the field of higher-order modes does not penetrate deeply into the metal nanorod.

**Figure 11:**
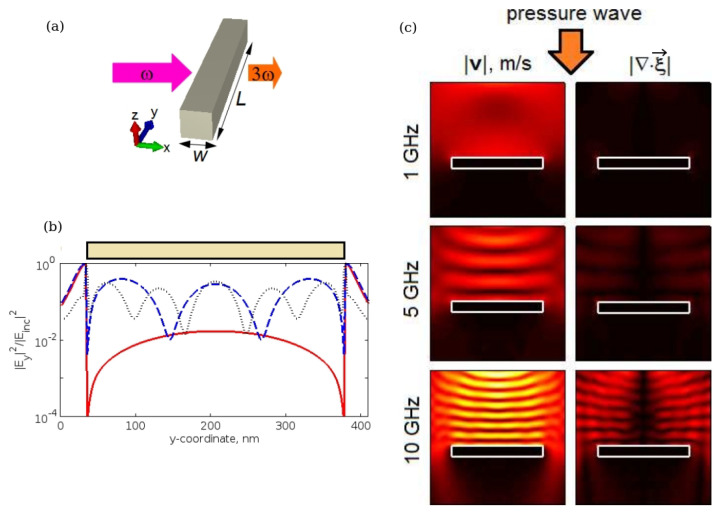
(**a**) Illustration of a numerical simulation configuration for investigation of the optical third harmonic generation in a 30 nm × 30 nm square cross-section silver nanorod immersed into water. A plane light wave polarised along the *y*-axis of the coordinate system is normally incident from the left. (**b**) Normalised simulated electric field intensity at the centre of the nanorod cross-section. The rectangle in the main panel schematically shows the length of the nanorod. The solid, dashed and dotted lines depict profiles of the fundamental mode, and the second and third higher-order modes, respectively. (**c**) Simulated spatial profiles of the acoustic velocity |**v**| (left column) and the divergence of the displacement field |$\nabla \cdot \vec{\zeta }$| for the acoustic frequency 1, 5 and 10 GHz. The white rectangle shows the contour of the nanorod. The black (yellow) colour denotes zero (maximum) for the profile intensity. The direction of the incident monochromatic acoustic pressure wave is indicated by the arrow. Note that the value of |**v**| in front of (behind) the nanorod increases (decreases) due to the well-known pressure doubling effect [118].

There are several approaches to the optical excitation of the higher-order plasmonic modes in a long nanorod. Firstly, one can tune the frequency of the incident light on the resonance frequency of a particular higher-order mode. This approach is relatively straightforward since tuneable laser sources are readily available. Alternatively, the geometry of a nanorod can be engineered to match a higher-order mode with a resonance frequency that coincides with the frequency of the available laser. However, the drawback of this approach is related to a tight confinement of the optical fields of the higher-order modes to the surface of a nanorod, which implies that the energy of such modes is not efficiently emitted into the electromagnetic far-field region. In turn, this means that the excitation of these modes by the incident light waves from the far-field is also insignificant. To overcome this inefficiency, the higher-order modes can be excited using a point-like quantum emitter of light (e.g., a quantum dot) located in the vicinity of a nanorod. However, such an excitation scheme would significantly increase the complexity of an experiment. An alternative viable approach could exploit nonlinear-optical properties [39] of the nanorod material [100]. In particular, using an intense laser beam to excite the fundamental mode of nanorod conditions can be created for the nonlinear generation of the second and third harmonics of the incident light [113,114,115]. In this case, the nanorod length should be so chosen so that the frequency of one or several of its higher-order modes coincides with the frequency of the nonlinearity-generated harmonics. The plausibility of this approach has been confirmed by numerical simulations, where a silver 340 nm × 30 nm × 30 nm nanorod with a square cross-section was investigated (Figure 11a,b) [20,116].

The main goal of studies reported in [20,116] was not only to numerically validate an earlier theoretical suggestion [117] of a plasmon-enhanced BLS effect in nanorods immersed in water or a biological fluid, but also to demonstrate that this approach could be used to generate an AFC. To achieve such a goal, a finite-difference time-domain model was developed [116], where Maxwell’s equations were solved simultaneously with the equations of nonlinear acoustics. While Maxwell’s equations describe the interaction of light with a metal nanorod and acoustically induced fluctuations of the refractive index of the surrounding liquid, the equations of nonlinear acoustics describe the interaction of acoustic waves with the nanorod.

The left column of Figure 11c shows the simulated fields of the sound velocity vector |**v**| in close proximity of a nanorod for the frequencies of the longitudinal acoustic pressure waves with frequencies $f_{a}$ = 1, 5 and 10 GHz. The contours of a 340 nm × 30 nm nanorod are shown by a white rectangle that also serves as the scalebar. Using these data, the displacement field $\vec{\zeta }$ of a particle from its equilibrium position due to the action of acoustic pressure waves was calculated. Subsequently, the divergence of the displacement field $\nabla \cdot \vec{\zeta }$ was computed and shown in the right column of Figure 11c. The amplitude of fluctuations of the dielectric permittivity of water δϵ caused by the propagating acoustic pressure wave is directly proportional to $\nabla \cdot \vec{\zeta }$ because a plane longitudinal acoustic wave propagating in the liquid results in alternating compression and rarefaction and corresponding density changes. The knowledge of the changes in the dielectric permittivity is required to numerically simulate the BLS from acoustic waves.

**Figure 12:**
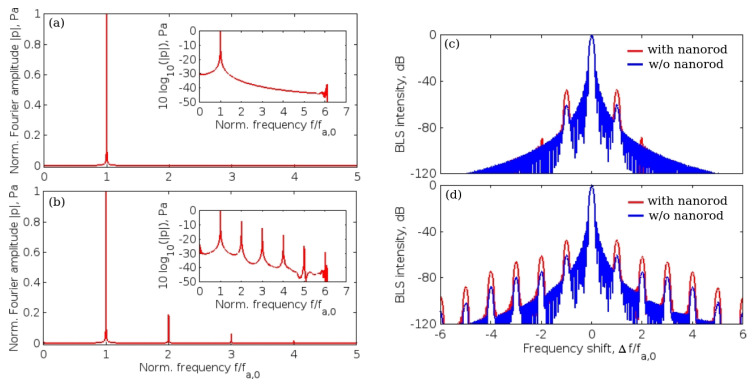
Normalised acoustic energy spectrum of the incident quasi-monochromatic acoustic pressure wave detected (**a**) before and (**b**) after it propagates through the bulk of water surrounding a nanorod showing the nonlinear generation of acoustic waves at harmonic frequencies of the incident acoustic pressure wave. The frequency is normalised to frequency $f_{a,0}$ of the incident wave. The peak amplitude of the incident wave is 5 MPa. The insets show the same spectra plotted in a decibel scale. (**c**,**d**) Simulated plasmon-enhanced intensity BLS signal produced from acoustic signals with the spectra shown in Panels (**a**,**b**). Note an approximately 35-fold enhancement due to plasmonic properties of a nanorod.

The simulated spectrum of the incident acoustic wave is shown in Figure 12a and its inset, where the same spectrum is plotted in the decibel scale. Only one peak at the normalised frequency $f/f_{a,0}=1$ can be seen confirming the monochromatic nature of this wave (since qualitatively the same result was obtained for kHz, MHz and GHz frequency range waves, the frequency was normalised to the frequency $f_{a,0}$ of the incident wave). However, when acoustic nonlinearity develops as a result of acoustic wave propagation in water, additional normalised frequency peaks appear in the spectrum at $f/f_{a,0}\;=\;2$ (quadratic nonlinearity effect), $f/f_{a,0}=3$ (cubic nonlinearity effect) and so on, see Figure 12b. In addition to the appearance of the wave harmonics, the nonlinear interaction leads to the creation of a mean drift (i.e., zero frequency) component that can be seen on a logarithmic scale.

The time-domain dependencies of the dielectric permittivity fluctuations δϵ(t) were also extracted from the numerical data and used in simulations of the plasmon-enhanced BLS effect, the results of which are presented in Figure 12c,d. Using the simulation data reported in Figure 12a, a typical BLS spectrum with the central Rayleigh peak and two weak side peaks shifted by the normalised frequency of the incident acoustic wave $\Delta f/f_{a,0}=\pm 1$ from it was found, see Figure 12c. Note that the amplitude of the shifted Brillouin peaks is significantly increased when a nanorod is present due to the plasmonic enhancement. The presence of a nanorod also leads to the appearance of the second order Brillouin peaks shifted by $\Delta f/f_{a,0}=\pm 2$ from the central peak. Most importantly, due to a strong acoustic nonlinearity present in Figure 12d the generation of an AFC is observed with the spectrum consisting of Brillouin peaks shifted by $\Delta f/f_{a,0}=\pm 1,\pm 2,\pm 3$ and so on with respect to the central peak. In the presence of a nanorod, the amplitude of all these peaks is increased due to its plasmonic behaviour. It was also demonstrated in the follow-up paper [20], where a more advanced model of the plasmon-enhanced BLS interaction was proposed, that all plasmon-enhanced Brillouin peaks are phase coherent and thus can be considered as an FC similar to a mode-locked laser OFC [1]. Finally, we note that qualitatively similar results were obtained for the values of $f_{a,0}$ in a wide range from several kHz to several GHz, which implies that the inter-peak distance of the AFCs discussed in this section can also lie in this wide frequency range.

## 6. Frequency Comb Generation Using Oscillations of Gas Bubbles in Liquids

### 6.1. Physical Origin of the Acoustic Nonlinearity of Gas Bubbles

From the discussion preceding this section, it becomes clear that strong acoustic nonlinearities can result in the generation of AFCs containing a large number of high-amplitude peaks. Similar conclusions can be drawn regarding the Kerr OFCs and other FC generation techniques relying on nonlinear optical phenomena. However, in the field of Kerr OFCs, there exist fundamental physical limitations that do not allow one to increase the intensity of the laser beam indefinitely to amplify nonlinear effects and thus to increase the number of peaks in the spectrum of the comb [8]. While special techniques and novel materials have been proposed to relax such limitations [119,120,121,122] either by optimising the conversion of the energy of the incident light into new frequency signals or minimising optical absorption losses, in general they cannot be removed completely.

However, recently it has been theoretically demonstrated that nonlinear optical effects could be effectively replaced by acoustic nonlinearities. For example, a change in the refractive index due to a propagating acoustic pressure wave can modulate an optical signal and, if the acoustic wave exhibits nonlinearities, these also become imprinted onto the modulated optical signal, effectively enabling the conversion of the acoustic nonlinearity into new optical signals [8]. Such a nonlinear acousto-optical interaction may generate additional optical frequencies.

This concept of a hybrid nonlinear acousto-optical interaction exploits the fact that acoustic nonlinearities are much stronger than their optical counterparts and that they can be induced using sound waves with a relatively low peak pressure amplitude (recall that the field of nonlinear optics was established only after the invention of powerful lasers since this is required to induce nonlinear optical effects). In this context, it is worth noting the so-called giant acoustic nonlinearities associated with the oscillations of gas bubbles in liquids [53,123,124,125,126,127,128,129,130,131,132,133,134,135]. When an acoustic pressure wave propagates through water, its initially sinusoidal waveform changes so that its initial monochromatic spectrum acquires higher harmonic frequencies, see Figure 12a,b. The more nonlinear the medium in which sound propagates, the stronger such a spectral enrichment. The degree of acoustic nonlinearity is often characterised by the acoustic parameter β=B/A, which is the ratio of coefficients *B* and *A* of quadratic and linear terms in the Taylor series expansion of the equation of state of a medium relating the thermodynamic pressure *p* in the medium with its density ρ, where subscript 0 denotes the values in the absence of sound [8,53]. The larger the value of β, the more nonlinear the medium, the stronger a distortion of the acoustic spectrum from the initial monochromatic state. For example, water with β=3.5 is more acoustically nonlinear than air with β≈0.7 [8], but the degree of nonlinearity is moderate in both media. However, when air bubbles are injected into water, the value of β increases to around 5000 [8,53]. The following qualitative discussion explains this fact.

Liquids are dense and have little free space between molecules, which leads to their low compressibility. In contrast, gases are easily compressible. When an acoustic wave propagating in water reaches a bubble, due to the high compressibility of air trapped in it its volume changes dramatically (see, e.g., [132]). This in turn results in large local acoustic wavefront deformations that result in strong variation of the initial acoustic spectrum. Consequently, whereas in bubble-free water one can observe the generation of five or so higher frequency harmonics of the incident sound, as seen from Figure 12b, in bubbly water up to 20 high-order acoustic harmonics can be generated for the acoustic wave with the same peak pressure amplitude, effectively forming AFC.


$$p=p\left(\rho \right)\approx p_{0}+A\frac{\rho -\rho_{0}}{\rho_{0}}+B\frac{{\left(\rho -\rho_{0}\right)}^{2}}{2\rho_{0}^{2}}+\cdots$$

### 6.2. Acoustic Frequency Comb Generation Using Oscillations of Multiple Gas Bubbles in Water

**Figure 13:**
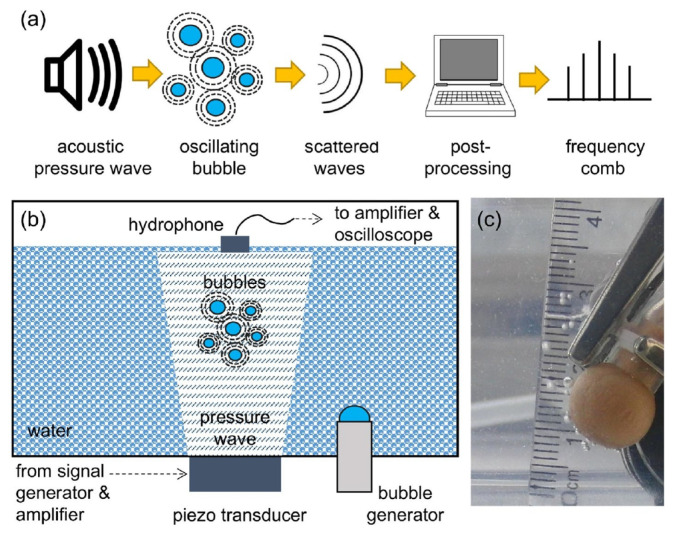
(**a**) Schematic diagram of the AFC generation using oscillations of gas bubbles in water. The oscillations are driven by a single-frequency ultrasound pressure wave. Acoustic waves scattered by the bubbles are recorded and post-processed to obtain a spectrum consisting of equidistant peaks. (**b**) Schematic of an experimental setup, where bubbles are created in a stainless steel tank using a bubble generator, the driving pressure wave is emitted by an ultrasonic transducer, and waves scattered by the bubbles are detected by a hydrophone. (**c**) Photograph of typical gas bubbles emitted by a bubble generator. Reproduced from [21] published by Springer Nature under the terms of the Creative Commons CC BY license.

**Figure 14:**
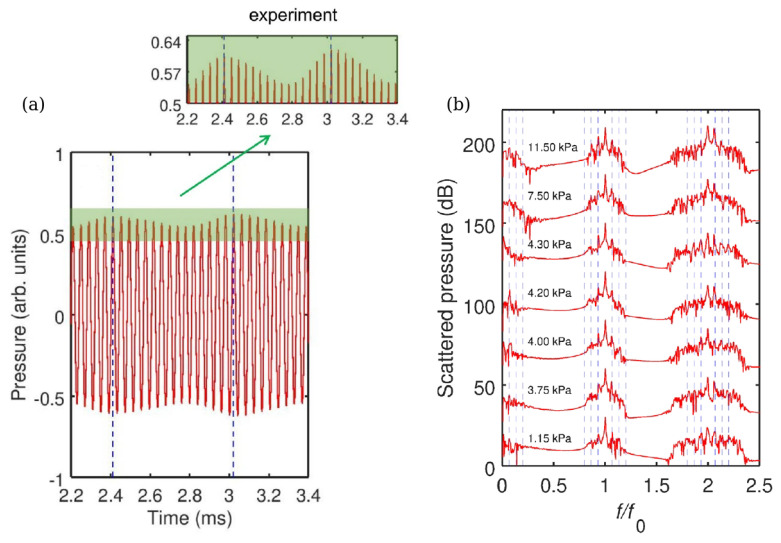
(**a**) Measured time-domain acoustic response of gas bubbles. The time between the vertical dashed lines is $\Delta T=1/f_{nat}\approx 0.6$ ms, where $f_{nat}$ is the natural frequency of the bubble oscillations (see [21] for details). The insets show a closeup of the waveforms and demonstrate the amplitude modulation. (**b**) Experimental AFC spectra obtained using gas bubbles in water insonated with an $f_{0}=24.6$ kHz sinusoidal signal of increasing pressure amplitude α = 1.15, 3.75, 4, 4.2, 4.3, 7.5 and 11.5 kPa. The scattered pressure values (in dB) are shown along the vertical axis with a vertical offset of 30 dB between the spectra. Reproduced from [21] published by Springer Nature under the terms of the Creative Commons CC BY license.

The ideas discussed above have been validated experimentally in [21], where a single-frequency ($f_{0}=24.6$ kHz) ultrasound wave irradiated several gas bubbles created in a water tank using a gas bubble generator, see Figure 13. A small (not exceeding 11.5 kPa) peak pressure amplitude of the driving ultrasound wave was deliberately chosen since, as discussed above, low-amplitude signals suffice to induce strong nonlinearities in liquid–gas mixtures. The generated bubbles had the equilibrium radii $R_{0}\approx 1.0\pm 0.5$ mm. However, since they interacted with each other during the oscillation driven by the ultrasound wave, a collective acoustic response typical of a small bubble cluster with an effective natural frequency [124,132] $f_{nat}\approx 1.7$ kHz was observed. Using high-speed imaging and following [136], it was estimated that the resulting cluster behaved similarly to a large single gas bubble with an equilibrium radius of 1.95 mm. Thus, since $f_{nat}$ is an order of magnitude lower than the frequency of the ultrasound wave, the oscillations of the cluster of bubbles resulted in a nonlinear generation of multiple ultraharmonic frequency peaks in the spectrum of the cluster’s acoustic response. The interaction of the so-generated acoustic waves with the noise-induced bubble oscillations at the natural frequency resulted in the amplitude modulation of the collective bubble response (Figure 14a), and the appearance of sidebands around the harmonic and ultraharmonic peaks (Figure 14b). These sideband structures can be used as AFCs.

**Figure 15:**
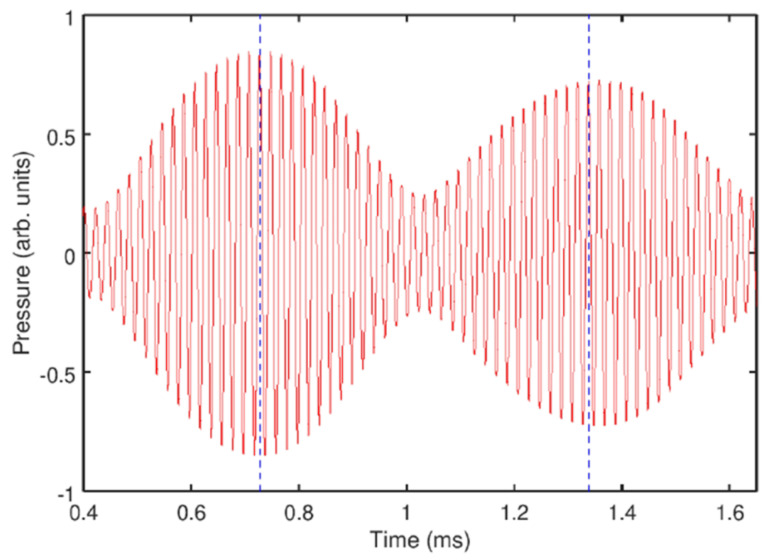
Measured acoustic bubble response corresponding to a sinusoidal driving pressure wave with the frequency $f_{0}=49.2$ kHz (twice the frequency in Figure 14a) and amplitude α=4.3 kPa. Reproduced from [21] published by Springer Nature under the terms of the Creative Commons CC BY license.

Several comments should be made to better explain this result. Firstly, in the temporal profile in Figure 14a, the amplitude modulation depth (the ratio of the modulation excursions to the amplitude of the unmodulated carrier wave) is smaller than 1. In some OFC technologies, where direct photodetection of the optical pulses is used to produce an electronic signal that follows the amplitude modulation of the pulse train, low modulation depth could pose technical challenges. However, this does not present a problem in the case of AFC using oscillating gas bubbles since the frequencies of the electronic signal of the AFC coincide with the frequencies of the driving pressure wave, which dramatically simplifies the characterisation of the comb. Secondly, in [21] the analysis of the experimental result was supported by a rigorous theoretical and computational modelling of gas bubble oscillations, and it was shown that both experimental time-domain signals and AFC spectra are in good qualitative agreement with the calculated ones. However, because the numerical model in [21] considered only a single oscillating gas bubble with an effective equilibrium radius of 1.95 mm, it was unable to reproduce some experimentally observed features, namely, the generation of another AFC spectrum centred at the second harmonic of the driving ultrasound wave. In fact, Figure 14b shows a sideband peak structure at 49.2 kHz (i.e., $f/f_{0}=2$) and peak ultrasound wave amplitude α=4.3 kPa. As shown in Figure 15, at this frequency, the amplitude modulation also gives rise to a train of pulses with the modulation period close to that of the natural bubble cluster oscillations, confirming that this signal can also be used as an AFC. Thirdly, a slight irregularity of the AFC peaks in Figure 14b was attributed to the Doppler effect associated with a translational motion of oscillating bubbles in the incident ultrasound field [133]. The size variation of the generated bubbles could also contribute to the comb peak imperfection. However, these deficiencies were not considered as prohibitive, which is demonstrated in the following section.

### 6.3. Spectrally Wide Acoustic Frequency Combs Generated Using Oscillations of Polydisperse Gas Bubble Clusters in Liquids

As with the other AFC generation techniques [9,55] discussed in this review article, in the experiments of [21] the number of the sideband peaks usable as an AFC is limited. Currently, this presents numerous technological challenges that shape research efforts in the field of AFCs (similar problems also exist in the field of OFCs [1,3]). For example, for many applications, the spectrum of an AFC has to span over an octave of bandwidth (i.e., the highest frequency in the FC spectrum has to be at least twice the lowest frequency). To achieve this, the spectrum of an AFC can be extended using one of the techniques developed, for example, for broadening the spectra of opto-electronic FCs [137] such as supercontinuum generation using nonlinear optical effects (as demonstrated above, the adoption of optical techniques in acoustics is possible because of the analogy between nonlinear optical processes in photonic devices and nonlinear acoustic processes in liquids containing gas bubbles). Furthermore, the theoretical analysis in [21] demonstrated that the number of peaks in a nonlinearly generated AFC and their magnitude can be increased by simultaneously decreasing the frequency and increasing the pressure of the ultrasound wave driving bubble oscillations.

As seen from Figure 14b, the shape of spectral peaks is slightly irregular and it could be argued that this artefact is associated with a translational motion of oscillating bubbles in the incident ultrasound field. Therefore, the question of long-term stability of AFC signals arises, which was comprehensively addressed in [22].

The interplay between radial bubble oscillations and their translational motion has been a subject of intensive research [131,135,138,139,140,141,142,143,144,145,146,147]. Most of these studies are based on the accepted models of spherical gas bubble oscillations [123,125,129] and consider Bjerknes forces [148] acting on oscillating bubbles. The primary Bjerknes force $F_{pB}$ is caused by the acoustic pressure field forcing a bubble [148,149], while the secondary Bjerknes force $F_{sB}$ arises between two or more interacting bubbles [133]. The secondary Bjerknes force between two gas bubbles is repulsive when the driving frequency lies between bubbles’ natural frequencies; otherwise, it is attractive [133,150,151].

In [22], an alternative strategy for broadening spectra of AFCs generated using gas bubble oscillations was suggested using polydisperse clusters consisting of mm-sized bubbles with equilibrium radii $R_{n0}=R_{10}/n$, where $R_{10}$ is the equilibrium radius of the largest bubble in the cluster and n=1,2,3,… is the total number of bubbles. Although clusters with other bubble size distributions could also be used in the proposed approach, it was shown that this specific ratio of equilibrium radii enables generating AFCs with a quasi-continuum of equally spaced peaks. Similarly to the experiment [21], in the analysis in [22] low-pressure ultrasound waves (up to 10 kPa) were considered and a numerical model of dynamics of multibubble clusters with translational motion developed in [133,144,152] was employed.

**Figure 16:**
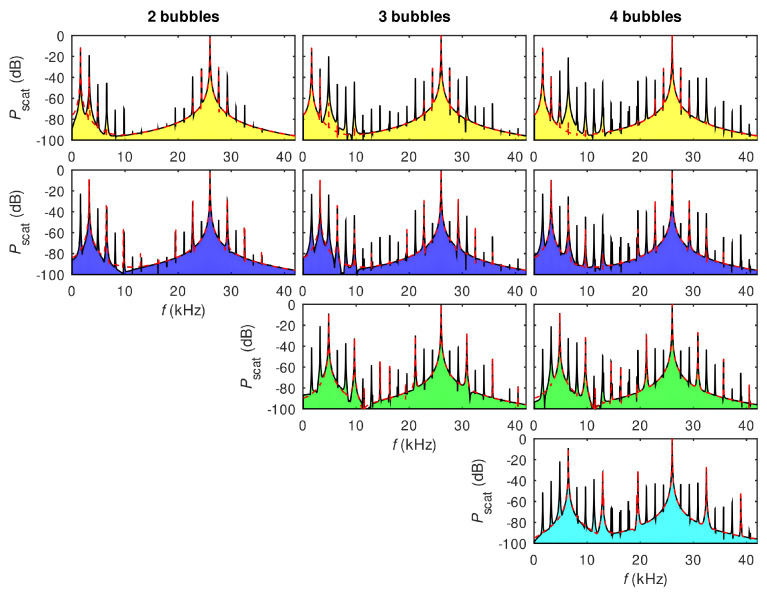
Columns (from left to right) show the AFC spectra produced by individual bubbles within clusters consisting of two, three and four bubbles with the equilibrium radii $R_{n0}=1.95/n$ mm, where *n* is the bubble index in the cluster. The number of panels in each column corresponds to the total number of bubbles. The red dashed lines in each panel show the spectra of individual non-interacting stationary bubbles with identical equilibrium radii. Computational parameters are given in [22]. Reproduced from [22]. Copyright 2021 by the American Physical Society.

Figure 16 shows the calculated spectra of the bubble clusters, where the number of rows in each column corresponds to the total number of bubbles in the cluster. Each column shows the spectrum of the pressure scattered by an individual bubble within the cluster. The inspection of panels within the same row from left to right reveals changes in the AFC peak structure caused by the addition of smaller bubbles to the cluster. For example, the four panels in the top row show that the number of equidistant peaks in the AFC spectrum produced by the largest bubble increases when smaller bubbles are added. This is because bubbles within a cluster are affected by the pressure waves scattered by their neighbours and thus their spectra include additional frequency peaks compared to the spectra of isolated non-interacting stationary bubbles of the same equilibrium radii (the dashed lines in Figure 16). Similarly, panels in the second row show the evolution of the AFC spectrum of the second largest bubble in the cluster, and so on. In all cases, the spectra exhibit a key feature of a pure AFC signal—the spectrum of the acoustic response of each bubble consists of a series of well-defined equally spaced peaks.

### 6.4. Temporal Stability of Bubble-Based Acoustic Frequency Combs

Numerical modelling reported in [22] demonstrated that the frequency of ultrasound wave driving bubble oscillations can be chosen in a wide spectral range above the natural oscillation frequency of bubbles in a cluster. This can greatly facilitate the generation and recording of stable AFC signals since at low pressure bubble clusters exhibit a regular behaviour for a longer time before their dynamics becomes affected by bubble aggregation. To examine the basic trends in the temporal stability of a bubble cluster, here we consider a system of just two interacting bubbles in liquid. This simplification allows reducing the complexity of the model while still accounting for essential physics of bubble interaction.

The accepted model of nonlinear oscillations of a single spherical gas bubble that does not undergo translational motion is the Keller–Miksis equation [129]. It takes into account the decay of bubble oscillations due to viscous dissipation and fluid compressibility. However, for mm-sized gas bubbles oscillating at 20–100 kHz frequencies in water being driven by low-pressure ultrasound waves with the amplitude of up to 10 kPa, the terms in the Keller–Miksis equation accounting for acoustic losses become negligible [22]. Thus, the Keller–Miksis equation effectively reduces to the classical Rayleigh–Plesset equation [123,125], which is written for a cluster consisting of *N* mm-sized gas bubbles not undergoing translational motion and being driven by low-pressure ultrasound waves as [131,135]

(1) $$R_{n}\frac{d^{2}R_{n}}{dt^{2}}+\frac{3}{2}{\left(\frac{dR_{n}}{dt}\right)}^{2}=\frac{1}{\rho }\left(P\left(R_{n},\frac{dR_{n}}{dt}\right)-P_{\infty }\left(t\right)\right)-P_{sn}\;,$$

where

(2) $$P_{n}\left(R_{n},\frac{dR_{n}}{dt}\right)=\left(P_{0}-P_{v}+\frac{2\sigma }{R_{n0}}\right){\left(\frac{R_{n0}}{R_{n}}\right)}^{3\kappa }-\frac{4\mu }{R_{n}}\frac{dR_{n}}{dt}-\frac{2\sigma }{R_{n}}\;.$$

The term accounting for the pressure acting on the *n*th bubble due to scattering of the incoming pressure wave by the neighbouring bubbles in a cluster is given by

(3) $$P_{sn}=\sum_{l=1,l\neq n}^{N}\frac{1}{d_{nl}}\left(R_{l}^{2}\frac{d^{2}R_{l}}{dt^{2}}+2R_{l}{\left(\frac{dR_{l}}{dt}\right)}^{2}\right)\;,$$

where $d_{nl}$ is the inter-bubble distance [131]. The pressure in a liquid far from the bubble is represented by $P_{\infty }\left(t\right)=P_{0}-P_{v}+\alpha \sin\left(\omega^{*}t\right)$ with the angular frequency $\omega^{*}=2\pi f$, where $P_{0}$ and $P_{v}$ are the air and vapor pressures, respectively. Parameters $R_{n0}$, $R_{n}\left(t\right)$, μ, ρ, κ, σ, α and *f* denote the equilibrium and instantaneous radii of the *n*th bubble in the cluster, the dynamic viscosity and the density of the liquid, the polytropic exponent of a gas entrapped in the bubble, the surface tension of a gas–liquid interface and the amplitude and the frequency of a driving ultrasound wave. Diffusion of the gas through the bubble surface is neglected.

To identify the main characteristics of nonlinear oscillations of interacting gas bubbles relevant to the generation of AFCs, an asymptotic analysis of Equation (1) is conducted after it is rewritten in the non-dimensional form using the equilibrium radius of the largest bubble in the cluster, $R_{10}$, and $1/\omega^{*}$ as the length and time scales, respectively, to introduce the non-dimensional quantities $r_{n}=R_{n}\left(t\right)/R_{10}$, $r_{l}=R_{l}\left(t\right)/R_{10}$ and $\tau^{*}=\omega^{*}t$ [135]. This results in

(4) $$\begin{array}{ccc} r_{n}r_{n}\prime \prime +\frac{3}{2}{{r_{n}}^{\prime }}^{2} & = & \left(M+\frac{W}{Q_{n}}\right){\left(\frac{Q_{n}}{r_{n}}\right)}^{K}-\frac{W}{r_{n}}-R\frac{{r_{n}}^{\prime }}{r_{n}}-M-M_{e}\sin\tau^{*}\\ & & -\sum_{\begin{array}{c} l=1\\ l\neq n \end{array}}^{N}\zeta_{nl}\left(r_{l}^{2}r_{l}^{\prime \prime }+2r_{l}{r_{l}^{\prime }}^{2}\right)\;, \end{array}$$

where $R=\frac{4\mu }{\rho \omega^{*}R_{10}^{2}}$, $W=\frac{2\sigma }{\rho \omega^{*2}R_{10}^{3}}$, $M=\frac{P_{0}-P_{v}}{\rho \omega^{*2}R_{10}^{2}}$, $M_{e}=\frac{\alpha }{\rho \omega^{*2}R_{10}^{2}}$, $\zeta_{nl}=\frac{R_{10}}{d_{nl}}$, K=3κ and $Q_{n}=\frac{R_{n0}}{R_{10}}$. Parameters R and W can be treated as inverse Reynolds and Weber numbers representing the viscous dissipation and surface tension effects, respectively. Parameter M characterises the ratio of the bubble’s natural and forced oscillation frequencies and $M_{e}$ is the measure of the ultrasound forcing [134]. Parameters $\zeta_{nl}$ and $Q_{n}$ are the inverse of the distance between the bubble centres and the bubble radius relative to that of the largest bubble in the cluster, respectively [135], and primes denote differentiation with respect to *t*. As discussed in [21,22,134], K=4 for air and for bubbles of sizes relevant to the AFC context but the maximum values of other parameters do not exceed $M=9.7\;\times \;10^{-4}$, $W=7.4\;\times \;10^{-7}$, $R=6.5\;\times \;10^{-6}$ and $M_{e}=9.9\;\times \;10^{-5}$. Therefore, the effects of water viscosity and surface tension on bubble oscillations are negligible and we set R=W=0 in what follows. Thus, ultrasonically forced bubble oscillations can be assumed as perfectly periodic when the driving frequency is much higher than any of the natural frequencies of the individual bubbles in the cluster (i.e., no resonances arise). This warrants using analysis similar to that of [21].

Consider a cluster consisting of two gas bubbles with the non-dimensional equilibrium radii $r_{n0}=Q_{n}$, n=1,2 ($Q_{1}\equiv 1$). Following [21,153] we look for the asymptotic solutions of Equation (4) in the form

(5) $$r_{n}=Q_{n}+\epsilon r_{n1}\left(\tau \right)+\epsilon^{2}r_{n2}\left(\tau \right)+\ldots \;,\;n=1,2\;,$$

where 0<ϵ≪1 is a parameter characterising the amplitude of bubble oscillations used to distinguish between various terms in the asymptotic series, $\tau =\omega \tau^{*}=\omega \omega^{*}t$ and $\omega =\sqrt{KM}$ is the Minnaert frequency [21,124] of the largest bubble in the cluster. At the first order of ϵ we obtain

(6) $$\begin{array}{ccc} {\ddot{r}}_{11}+r_{11}+Q_{2}^{2}\zeta_{12}{\ddot{r}}_{21} & = & p\sin(\Omega \tau )\;, \end{array}$$

(7) $$\begin{array}{ccc} {\ddot{r}}_{21}+\frac{1}{Q_{2}^{2}}r_{21}+\frac{\zeta_{12}}{Q_{2}}{\ddot{r}}_{11} & = & \frac{p}{Q_{2}}\sin\left(\Omega \tau \right)\;. \end{array}$$

where overdots denote differentiation with respect to τ, $\left(M_{e}/\omega^{2}\right)\sin\tau^{*}\equiv -\epsilon p\sin\left(\Omega \tau \right)$ and Ω≡1/ω≫1. At $O(\epsilon^{2})$, the equations become:

(8) $$\begin{array}{ccc} & {\ddot{r}}_{12}+r_{12}+Q_{2}^{2}\zeta_{12}{\ddot{r}}_{22}=\frac{K+1}{2}r_{11}^{2}-\frac{3}{2}{\dot{r}}_{11}^{2}-r_{11}{\ddot{r}}_{11}-2Q_{2}\zeta_{12}\left({\dot{r}}_{21}^{2}+r_{21}{\ddot{r}}_{21}\right)\;, & \end{array}$$

(9) $$\begin{array}{ccc} & {\ddot{r}}_{22}+\frac{1}{Q_{2}^{2}}r_{22}+\frac{\zeta_{12}}{Q_{2}}{\ddot{r}}_{12}=\frac{K+1}{Q_{2}^{2}}r_{21}^{2}-\frac{3}{2Q_{2}}{\dot{r}}_{21}^{2}-\frac{1}{Q_{2}}r_{21}{\ddot{r}}_{21}-2\frac{\zeta_{12}}{Q_{2}}\left({\dot{r}}_{11}^{2}+r_{11}{\ddot{r}}_{11}\right)\;. & \end{array}$$

Following [21], we write the random initial conditions as $r_{1}\left(0\right)=1+\epsilon a$, $r_{2}\left(0\right)=Q_{2}+\epsilon b$, $\dot{r_{1}}\left(0\right)=\epsilon c$ and $\dot{r_{2}}\left(0\right)=\epsilon d$ which results in

(10) $$r_{11}\left(0\right)=a\;,\;r_{21}\left(0\right)=b\;,\;{\dot{r}}_{11}\left(0\right)=c\;,\;{\dot{r}}_{21}\left(0\right)=d\;.$$

Subsequently, we obtain the leading order solutions

(11) $$\begin{array}{ccc} r_{11}\left(\tau \right) & = & B_{1}\sin\Omega \tau +C_{11}\cos\left(\omega_{1}^{\prime }\tau \right)+C_{12}\sin\left(\omega_{1}^{\prime }\tau \right)\\ & & +C_{21}\cos\left(\omega_{2}^{\prime }\tau \right)+C_{22}\sin\left(\omega_{2}^{\prime }\tau \right)\;, \end{array}$$

(12) $$\begin{array}{ccc} r_{21}\left(\tau \right) & = & B_{2}\sin\Omega \tau +\frac{1-{\omega_{1}^{\prime }}^{2}}{{\omega_{1}^{\prime }}^{2}Q_{2}^{2}\zeta_{12}}\left(C_{11}\cos\left(\omega_{1}^{\prime }\tau \right)+C_{12}\sin\left(\omega_{1}^{\prime }\tau \right)\right)\\ & & +\frac{1-{\omega_{2}^{\prime }}^{2}}{{\omega_{2}^{\prime }}^{2}Q_{2}^{2}\zeta_{12}}\left(C_{21}\cos\left(\omega_{2}^{\prime }\tau \right)+C_{22}\sin\left(\omega_{2}^{\prime }\tau \right)\right)\;, \end{array}$$

(13) $$\begin{array}{ccc} \omega_{1,2}^{\prime } & = & \sqrt{\frac{2}{Q_{2}^{2}+1\pm \sqrt{{\left(Q_{2}^{2}-1\right)}^{2}+4Q_{2}^{3}\zeta_{12}^{2}}}}\;. \end{array}$$

These frequencies depend on the inverse inter-bubble distance $\zeta_{12}$ [133,138,154]. Considering a particular case of $Q_{2}=\frac{1}{2}$, as expected, for non-interacting distant bubbles with $\zeta_{12}\to 0$, we obtain $\omega_{1}^{\prime }\to \omega_{10}^{\prime }=1$ and $\omega_{2}^{\prime }\to \omega_{20}^{\prime }=2$. In general, the leading order bubble response will always contain three distinct frequencies: two bubbles’ natural frequencies $\omega_{1,2}^{\prime }$ and the driving ultrasound frequency Ω.

Coefficients $B_{i}$ and $C_{ij}$, i,j=1,2 in Equations (11) and (12) depend on $\zeta_{12}$, Ω and *p* and can be obtained for arbitrary initial conditions (10). However, their expressions are too long to be given here explicitly. We only state that they demonstrate that the magnitude of the $\omega_{1}^{\prime }$ peak is greater than that of $\omega_{2}^{\prime }$ in the spectrum of bubble 1 and vice versa and the amplitude of the peak corresponding to the frequency of a neighbouring bubble decreases with the distance between them.

Analysis of Equations (8) and (9) can be performed following the procedure outlined in [21]. However, it suffices here just to note that the right-hand sides of these equations contain quadratic terms involving $r_{11}$ and $r_{12}$ and their derivatives. Therefore, in addition to the harmonic components with frequencies $\omega_{1,2}^{\prime }$, solutions of Equations (8) and (9) will include steady and periodic terms with frequencies equal to all possible pair-wise sums and differences of $\omega_{1,2}^{\prime }$ and Ω: $\omega_{1,2}^{\prime }\pm \omega_{2,1}^{\prime }$, $\Omega \pm \omega_{1,2}^{\prime }$, $2\omega_{1,2}^{\prime }$ and 2Ω. In the AFC context, the frequency spectrum centred at Ω is important, that is spectral lines $\Omega -\omega_{1}^{\prime }\left(1+\Delta \right)$, $\Omega -\omega_{1}^{\prime }$, Ω, $\Omega +\omega_{1}^{\prime }$, $\Omega +\omega_{1}^{\prime }\left(1+\Delta \right)$, where

$$\Delta =\frac{\omega_{2}^{\prime }-\omega_{1}^{\prime }}{\omega_{1}^{\prime }}\approx \frac{1}{Q_{2}}-1+\frac{1}{2}\frac{1+Q_{2}^{2}}{1-Q_{2}^{2}}\zeta_{12}^{2}=1+\frac{5}{6}\zeta_{12}^{2}\;.$$

For an ideal AFC, Δ=1, which is the case in the limit $\zeta_{12}\to 0$ of distant bubbles. It also follows from the above expression that to keep the spectral non-uniformity of a bubble-based AFC within 5% it is sufficient to ensure that no bubbles in a cluster approach each other closer than about two diameters of the largest bubble.

To assess the robustness of a bubble-based AFC, we show that the attractive secondary Bjerknes force acting between two distant bubbles is negligible in the AFC conditions. The expression for such a force arising is given, for example, by Equation (2.5) in [133]. Scaled with $\rho {\omega^{*}}^{2}R_{10}^{4}$ and with formal parameter ϵ set to 1, it reads

(14) $$F_{sB}^{\prime }=-4\pi \zeta_{12}^{2}Q_{2}^{3}\omega^{2}\langle r_{11}{\ddot{r}}_{21}\rangle \;,$$

where the angle brackets denote time averaging. Substituting expressions (11) and (12) into Equation (14) and keeping only the largest terms in each group in the limits of Ω→∞ and $\zeta_{12}\to 0$ leads to

(15) $$F_{sB}^{\prime }=\frac{2\pi \zeta_{12}^{2}Q_{2}^{3}\omega^{2}}{{\left(Q_{2}^{2}-1\right)}^{2}+4Q_{2}^{3}\zeta_{12}^{2}}\left(F_{sBn}^{\prime }+F_{sBu}^{\prime }+F_{sBun}^{\prime }\right)\;,$$

where

$$\begin{array}{ccc} F_{sBn}^{\prime } & = & \left(Q_{2}^{2}-1\right)\left(b^{2}-Q_{2}\left(a^{2}+c^{2}-Q_{2}d^{2}\right)\right)\zeta_{12}+2\left(ab\left(Q_{2}^{2}+1\right)+2cdQ_{2}^{2}\right)Q_{2}\zeta_{12}^{2}\;,\\ F_{sBu}^{\prime } & = & \frac{M_{e}^{2}}{\omega^{4}\Omega^{2}}\left[\frac{{\left(Q_{2}^{2}-1\right)}^{2}}{Q_{2}}+\left(5-2Q_{2}^{2}+5Q_{2}^{4}\right)\zeta_{12}^{2}\right]\;,\\ F_{sBun}^{\prime } & = & 2\frac{M_{e}Q_{2}}{\omega^{2}\Omega }\left[\left(Q_{2}^{2}-1\right)\left(d-c\right)\zeta_{12}+\left(Q_{2}^{2}+1\right)\left(d+cQ_{2}\right)\zeta_{12}^{2}\right] \end{array}$$

are the Bjerknes force components due to natural bubble oscillations, ultrasound forcing and the interaction between the two, respectively. By definition, $Q_{2}<1$ and in the reference experiment [21], $M_{e}/\omega^{2}∼2.6\;\times \;10^{-2}$, Ω∼16 and $\zeta_{12}^{2}≲0.04$. Therefore, we conclude that the secondary Bjerknes force is small at the typical driving frequencies used in the generation of bubble-based AFCs away from bubble resonances. This provides an opportunity for measuring the acoustic bubble response and recording the resulting signals for AFC applications before bubble oscillations become affected by their aggregation.

## 7. Acoustic Frequency Comb Generation Using Vibrations of Liquid Drops

In this section, we discuss similarities between nonlinear acoustic properties of liquid drops and gas bubbles in the context of AFC generation. The natural tendency to minimisation of a surface tension energy explains the spherical shape of undisturbed bubbles. The surface tension also defines the shape of liquid drops that, despite being easily deformable, tend to assume a spherical shape also. Subsequently, similar to gas bubbles, liquid drops can oscillate in response to an acoustic forcing and develop nonlinear behaviour analogous to that of oscillating gas bubbles [8,68,155].

More specifically, natural oscillations of a liquid drop are driven by capillary forces competing with inertia of the liquid [156]. This leads to rich physical behaviours that warrant considering liquid drop studies as an independent and presently burgeoning field of research [23,157,158,159,160,161,162,163,164,165,166,167,168,169,170,171]. An important setup in this field is parametrically excited waves on the surface of vertically vibrated liquid drops, the phenomenon originally observed on the surface of a liquid layer in 1831 by Faraday. Such waves have become a paradigmatic example of a nonlinear wave system that exhibits complex dynamics including periodic [172], quasi-periodic [173,174,175] and chaotic behaviour [176,177,178,179]. Recent studies have opened new frontiers for potential applications of Faraday waves extending beyond fluid dynamics. For example, in photonics, they have been used to generate a special kind of OFC [180].

Physical processes behind the excitation of Faraday waves in liquids are conceptually similar to mechanical vibrations used to generated phononic FCs (Section 4). Therefore, it is plausible to assume that Faraday waves in liquids can also be employed to generate AFCs. However, before we discuss the relevant results, we assess several key characteristics of potential Faraday wave-based FCs.

In liquids, the restoring force arises from surface tension [156]. The speed of capillary waves in a liquid is three orders of magnitude smaller than that of acoustic pressure waves [8]. As a result, the frequency of capillary oscillations is about three orders of magnitude smaller than that of an acoustic wave mode. These observations imply that the FC generation using Faraday waves in liquids would result in FC spectra with a small inter-peak distance of the order of several tens of Hz that can find applications, for example, in underwater acoustics [5]. Moreover, capillary oscillations of liquid drops can be excited using non-mechanical techniques such as electrowetting, where a sessile drop oscillates when an alternating voltage is applied to it via a contact electrode. The oscillations result from a time-varying electrical force acting on the three-phase contact line. They lead to resonances that occur at certain frequencies of the applied alternating electric signal [181]. While for mm-sized liquid drops, such resonance frequencies can be in the range of from 30 to 300 Hz, using room-temperature liquid metal alloy nanodrops one can increase them to several GHz [182], which is beneficial for AFC applications. We will return to the discussion of the relevant physical properties of liquid metal drops in Section 7.5.

**Figure 17:**
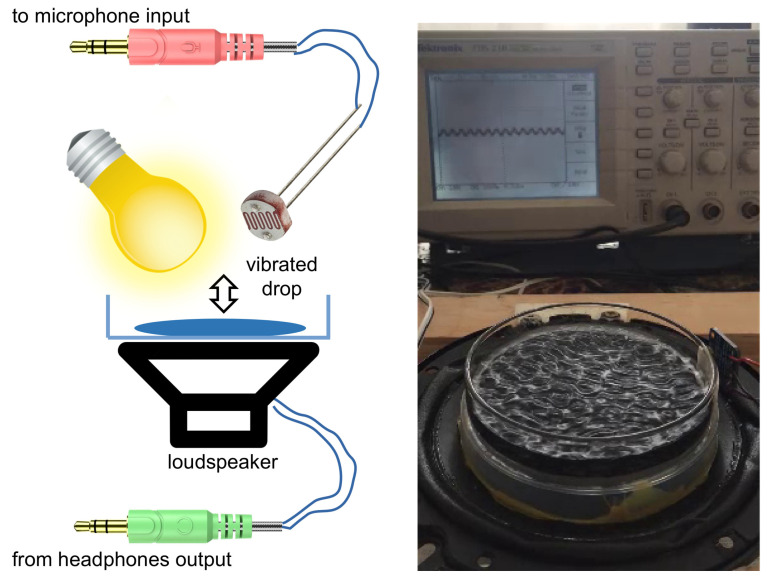
(**Left**) Schematic of a technically simple experimental setup consisting of a low-frequency loudspeaker connected via a power amplifier to the headphone output of a laptop computer. A Petri dish is glued to the loudspeaker. An audio signal is produced by a tone generator. The fluid surface is illuminated by a light source and Faraday waves are detected using a photoresistor that is connected either to an oscilloscope or to the microphone input of the laptop computer with a pre-installed audio signal processing software. (**Right**) Photograph of Faraday waves on the surface of water contained in a Petri dish glued to a vibrating loudspeaker.

However, relatively low viscosity of common liquids such as water implies that Faraday surface waves can be excited using low-amplitude vertical vibrations produced by inexpensive and readily available components such as loudspeakers and piezo-electric transducers (Figure 17) that are much simpler than any equipment used in electrowetting experiments [181]. As discussed in [183,184,185], enhancements of the basic setup used to investigate Faraday waves in oscillating drops also include common elements such as diode lasers, measurement-grade photodetectors and accelerometers, which nevertheless revealed a number of intriguing physical processes that can also help understand the behaviour of smaller drops oscillating at much higher frequencies [186].

### 7.1. Experimental Demonstration of AFC Generation Using Faraday Waves

To demonstrate the plausibility of AFC generation using Faraday waves in liquids, in the experiment of [24] a red laser diode (650 nm wavelength, 0.5 mW power) with a highly divergent beam profile was used as the source of light. A loudspeaker (3 W, 20 Hz–15 kHz) was driven via an audio amplifier by a pure sinusoidal signal f=100 Hz. One end of a cardboard cylinder (height 20 cm, radius 4.2 cm) was fixed above the loudspeaker and the other end was covered with a 0.5 mm thick black Teflon membrane. A pancake-like drop of pure alcohol (95% *v*/*v* ethanol) was placed on top of the membrane. The thickness of the liquid layer was 2 mm. A photodetector with a frequency response covering the entire frequency range of a loudspeaker was used to receive light reflected from the liquid surface.

**Figure 18:**
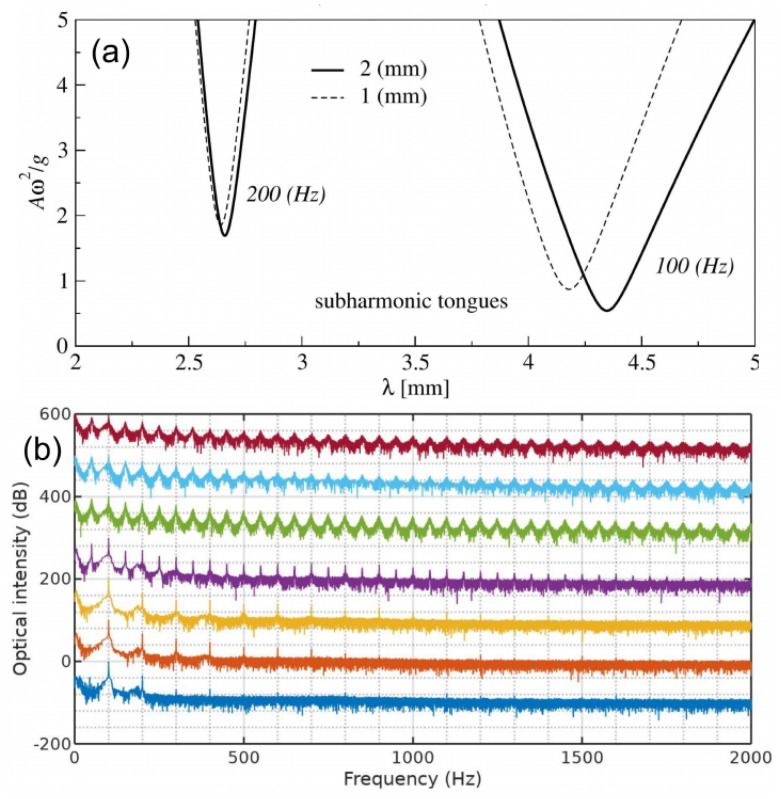
(**a**) Stability diagram for the h=2 mm and h=1 mm deep ethanol layers showing two subharmonic Faraday tongues. The oscillation wave frequency is $f_{F}=50$ Hz, half of the vertical vibration frequency f=100 Hz. (**b**) Experimental optical spectra for seven gradually increasing (from bottom to top) amplitudes of the vibration signal with f=100 Hz. The depth of the ethanol layer is h=2 mm. All spectra are vertically offset by 100 dB. The three lowest spectra are dominated by the vibration frequency f=100 Hz and its higher-order harmonics nf (n=2,3,⋯) that appear due to nonlinear acoustic effects in ethanol. The fourth and so on spectra are dominated by the Faraday wave at $f_{F}=50$ Hz and its higher-order harmonics. Note that all peaks have a characteristic triangular shape (see the main text). The equal spacing between them allows using the spectra as AFCs. Reproduced from [24] with permission of SPIE and the authors of the publication.

The classical nonlinear standing Faraday waves appear on the surface of a horizontally extended fluid in a vertically vibrating container. When the normalised vibration amplitude $A\omega^{2}/g$ (ω=2πf, *f* is the vibration frequency and *g* is the gravity acceleration) exceeds the critical value, a flat fluid surface becomes unstable and subharmonic surface waves oscillating at the frequency f/2 are formed. Figure 18a shows the stability diagram for h=2 mm and h=1 mm deep ethanol layers, where the so-called subharmonic Faraday tongues that correspond to the neutral stability of perturbation with wavelength λ and half of the driving frequency f/2 can be seen [63]. The flat fluid surface is linearly unstable above the boundary of the tongues. For f=100 Hz (200 Hz) and h=2 mm, Faraday wave wavelength is λ=4.34 mm (2.66 mm) at $A\omega^{2}/g=0.53$ (1.7). For f=100 Hz and h=2 mm, a high-speed camera was used to estimate wavelength λ≈4.5 mm, which is in good agreement with the theoretical prediction.

However, in the experiment of [24], the fluid forms a pancake-shaped liquid drop on the surface of a solid membrane. The formation of Faraday waves in this case is qualitatively different. When such a drop is vertically vibrated with a small amplitude, the capillary surface waves are excited at the edge of a drop, i.e., at the contact line between the fluid and the membrane. Similarly to a classical damped harmonic oscillator, the frequency of the excited waves is identical to the vibration frequency *f*. This result is confirmed in Figure 18b (see the three lowest spectra). As the amplitude increases remaining below the critical value, new peaks appear at the harmonic frequencies nf (n=2,3,…). In agreement with the theory, these peaks are due to nonlinear-acoustic effects in ethanol triggered by the incident acoustic wave f=100 Hz. The onset of Faraday waves in finite-volume liquid drops is associated with the period-doubling bifurcation [170]. When the amplitude of the 100 Hz wave reaches the Faraday instability threshold, surface Faraday waves are excited at $f_{F}=f/2=50$ Hz. The nonlinearity of these waves is so strong that one can observe many higher-order harmonics $nf_{F}$. The height of the peaks associated with Faraday waves is much larger than that of driving 100 Hz frequency of the acoustic wave and it has a characteristic triangular shape. This shape is a signature of extreme nonlinearities observed in fluid-mechanical systems [177] leading to the formation of capillary rogue waves [187], oscillons [176] and solitons [188].

**Figure 19:**
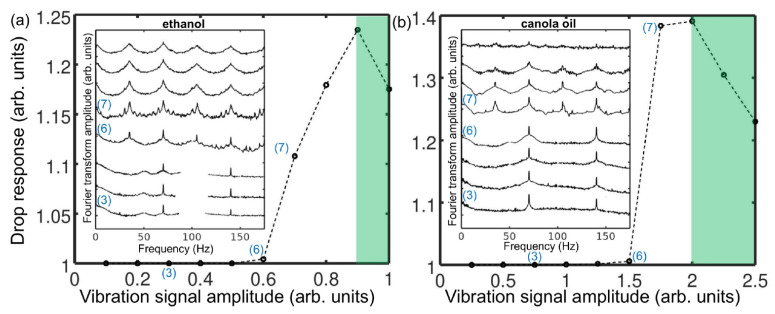
(**a**) Experimental average response of an ethanol drop subjected to vertical vibration at 70 Hz plotted as a function of the vibration amplitude. The inset shows the power spectra obtained by Fourier-transforming the measured signals. The parenthetical labels relate the spectra to the experimental points in the main panel. (**b**) Experimental average response of a canola oil drop subjected to vertical vibration at 70 Hz plotted as a function of the vibration amplitude. The modulation sidebands (the spectrum label 7) are present in the ethanol drop spectra and absent in the spectra of a canola oil drop. The shaded regions in the main panels correspond to the chaotic oscillations, resulting in strong diffuse scattering leading to a decrease in the optical intensity of the detected signal. Reproduced from [23]. Copyright 2019 by the American Physical Society.

To understand the physics behind the changing peak shape, a more detailed experiment was conducted in [23] using smaller pancake-like drops of ethanol and canola oil (Figure 19). The observations revealed a number of peculiarities relevant to the generation of AFC. Their theoretical explanation is given in the following section.

### 7.2. Existence Conditions for Faraday-Wave-Based Acoustic Frequency Combs

Here, we summarise the results of the analysis reported in [23] with a special focus on the physical conditions that are favourable for the generation of Faraday wave-based AFCs. To start with, we note that modelling the dynamics of a liquid drop that rests on a solid plate requires a correct description of the contact line motion. To avoid the well-known hydrodynamic singularities at the true contact line [189], a standard regularisation method is used that is based on the assumption that a molecularly thin precursor film exists beneath the drop and covers the entire area of the solid plate [156]. In this case, the equilibrium contact angle can be determined by balancing the pressure in the precursor film and in the drop. For small contact angles, the total pressure can be written in terms of the drop thickness h(x,y) [156]

(16) $$\begin{array}{c} P=-\sigma \left(\partial_{xx}+\partial_{yy}\right)h+\rho gh-\Pi \left(h\right)\;, \end{array}$$

where σ and ρ are the surface tension and the fluid density and Π(h) is the disjoining pressure that describes the long-range van der Waals forces giving rise to the formation of the precursor film. In general, the function Π(h) that allows the existence of a steady drop with a non-zero contact angle is given by [190]

(17) $$\begin{array}{c} \Pi \left(h\right)=\frac{B}{h_{\infty }^{n}}\left(\frac{h_{\infty }^{n}}{h^{n}}-\frac{h_{\infty }^{m}}{h^{m}}\right)\;, \end{array}$$

where *B* is a constant, $h_{\infty }$ is the thickness of the precursor film and m,n are some integer numbers. Following [158,170] and choosing n=6 and m=3, the disjoining pressure Equation (17) can be written in terms of the Hamaker constant $A_{H}$, a constant that accounts for van der Waals interaction between two materials, as

(18) $$\begin{array}{c} \Pi \left(h\right)=\frac{A_{H}}{h^{3}}\left(\frac{h_{\infty }^{3}}{h^{3}}-1\right)\;. \end{array}$$

The equilibrium contact angle θ≪1 is related to $A_{H}$ and $h_{\infty }$ via [190]

(19) $$\begin{array}{c} A_{H}\approx \frac{5h_{\infty }^{2}}{3}\sigma \theta^{2}\;. \end{array}$$

Subsequently, we adopt a simplified version of the model proposed in [170], where we only take into account long-wave deformations of the liquid–gas interface assuming a quadratic dependence of the horizontal fluid velocity u(x,y,z) on the vertical coordinate *z*

(20) $$\begin{array}{c} u=\Phi \left(\frac{z^{2}}{2}-hz\right)\;. \end{array}$$

This expression satisfies the boundary conditions at the solid plate u(z=0)=0 and at the liquid–gas interface $\partial_{z}u\left(z=h\right)=0$ for some arbitrary function Φ(x,y). The flow field across the layer $q=\int_{0}^{h}u\;dz$ can be expressed in terms of Φ

(21) $$\begin{array}{c} q=-\Phi \frac{h^{3}}{3}\;. \end{array}$$

The Navier–Stokes equation in the long-wave approximation is

(22) $$\begin{array}{c} \rho \left(\partial_{t}u+\nabla \left(u^{2}\right)+\partial_{z}\left(uw\right)\right)=\mu \partial_{zz}u-\nabla P\;, \end{array}$$

where μ is the dynamic viscosity, *w* is the vertical component of the velocity and *P* is the pressure. Integrating Equation (22) over *z* and using the kinematic boundary condition $\partial_{t}h+\left(u\cdot \nabla h\right)=w$, we obtain

(23) $$\begin{array}{ccc} \rho \left[\partial_{t}q+\frac{6}{5}\nabla \cdot \left(\frac{q⊗q}{h}\right)\right] & = & -\frac{3\mu q}{h^{2}}-h\nabla P,\\ \partial_{t}h+\left(\nabla \cdot q\right) & = & 0\;, \end{array}$$

where q⊗q denotes the matrix product.

When a drop is supported by a solid plate that vibrates vertically with amplitude $A_{0}$ and frequency Ω, Equations (23) are valid in the frame co-moving with the plate. The pressure *P* is taken from Equation (16) with *g* replaced by g(1+acosΩt), where $a=A_{0}\Omega^{2}/g$ is the dimensionless vibration amplitude. The validity of system Equation (23) is restricted to small contact angles and small variations of the drop height *h*. In addition, the characteristic horizontal deformation wavelength λ must be larger than the length of the viscous boundary layer $l=\sqrt{2\mu /(\rho \Omega )}$ associated with the vibration frequency Ω [191]. In what follows, we consider the simplest possible case of a one-dimensional liquid drop, whose dynamics is described by Equations (23) with h(x,t) and one-dimensional fluid flux q(x,t).

### 7.3. Linear Response and Higher Harmonics

In the absence of external driving, i.e., when a=0, Equations (23) admit a steady-state solution $h_{s}\left(x\right)$, characterised by zero fluid flux q(x,t)=0. In an unbounded domain, this solution satisfies $\lim_{x\to \pm \infty }h_{s}\left(x\right)=h_{\infty }$ and corresponds to an equilibrium liquid drop resting on the precursor film of thickness $h_{\infty }$. Drops with a sufficiently large volume (excluding the volume of the precursor film) are flattened by the gravity, resembling a cross-section of a pancake. The height $h_{0}$ of the drop is much larger than the precursor film thickness $h_{\infty }$ and the volume of the drop determines its width *W*.

In response to a weak vibration a≪1, the drop develops the so-called harmonic small-amplitude capillary waves on its surface, whose oscillation frequency is identical to the vibration frequency Ω. The exact form of the temporal response of the drop to a weak external vibration can be determined by linearising Equation (23) about a flat upper cap of the drop $h=h_{0}+\tilde{h}\left(x,t\right)$, where $\tilde{h}\ll h_{0}$ is a small deformation amplitude. As was shown in [23], the resulting linearised equation is given by the damped-driven Mathieu equation

(24) $$\begin{array}{c} \partial_{tt}\tilde{h}+\frac{3\mu }{\rho h_{0}^{2}}\partial_{t}\tilde{h}+h_{0}\left(\frac{\sigma }{\rho }\partial_{x}^{4}+g\left(1+a\cos\Omega t\right)\partial_{x}^{2}\right)\tilde{h}=p_{e}\left(x,t\right)\;, \end{array}$$

where $p_{e}\left(x,t\right)$ denotes the excess pressure, generated by the left and the right oscillating drop edges.

Next we observe that $p_{e}\left(x,t\right)$ oscillates with the driving frequency Ω and expand $\tilde{h}=a{\tilde{h}}^{\left(0\right)}+a^{2}{\tilde{h}}^{\left(1\right)}+a^{3}{\tilde{h}}^{\left(2\right)}+\ldots$. The analysis conducted in [23] based on Equation (24) reveals that the leading order response ${\tilde{h}}^{\left(0\right)}$ is harmonic, i.e., ${\tilde{h}}^{\left(0\right)}$ oscillates with the driving frequency Ω. However, the higher-order terms ${\tilde{h}}^{\left(1,2,\ldots \right)}$ contain higher-order harmonics of the driving signal. It is therefore evident that, already in the linear regime, the temporal spectrum of the drop response can be used as a frequency comb with delta-like peaks at frequencies nΩ, (n=1,2,3,…).

### 7.4. Nonlinear Response and the Amplitude Modulation

To investigate the nonlinear response of the drop and to further identify a regime that would be favourable for the AFC generation, we employ the continuation method, where Equations (23) are solved numerically for gradually varying (increasing or decreasing) values of the vibration amplitude *a*: $a_{n}=n\Delta$, n=0,1,2,3,… with a fixed step Δ=0.0015. For each value of *n*, Equations (23) are integrated over the time interval that is equivalent to 1000 oscillation cycles. The solution from the (n−1)th run is taken as the initial condition for the *n*th run. To artificially recreate the experimental conditions reported in [23], a small-amplitude white noise ξ(x,t) is added to the second of Equations (23).

**Figure 20:**
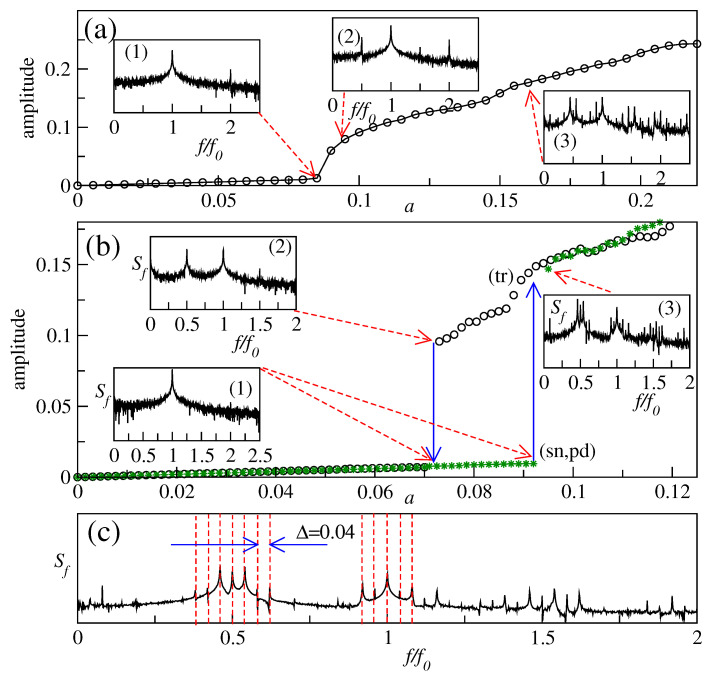
Average response amplitude of an ethanol drop vibrated at the frequencies (**a**) *f* = 21 and (**b**) 28 Hz as a function of the gradually increasing amplitude *a*. Harmonic waves lose their stability (**a**) via a supercritical period-doubling bifurcation (pd) at a=0.08 and (**b**) via a torus bifurcation at a=0.09. The modulational instability sets in via a torus bifurcation (tr) on the subharmonic branch. At f=28 Hz, one observes the formation of a hysteresis loop consisting of gradually increasing (asterisk) and gradually decreasing (circle) branches, where sn and tr correspond to the saddle-node and torus bifurcations, respectively. (**c**) Close-up of spectrum $S_{f}$ (3) in panel (**b**) in the regime of the developed modulational instability. The dashed vertical lines highlight the location of equally distant delta peaks with the scaled inter-peak distance of $f/f_{0}=\Delta =0.04$.

The deformation δh(t) of the drop surface at x=0 and its power spectrum $S_{f}$ are used to characterise the temporal response of a fixed-volume ethanol drop vibrated with different frequencies *f*. In Figure 20, the amplitude of δh(t) in the units of the drop height $h_{0}$ is shown as a function of *a* for a fixed-volume ethanol drop vibrated at 21 Hz (Figure 20a) and 28 Hz (Figure 20b).

The characteristics of the drop response are highly sensitive to the relationship between the Faraday wavelength $\lambda_{F}$ and the horizontal size of the drop *W*. Since in the relevant experiment the volume of the drop was fixed, in the model the vibration frequency *f* was varied, resulting in a change of the dimensional wavelength of Faraday waves. Thus, at the vibration frequency 21 Hz, we observe the onset of the sub-harmonic Faraday waves via a super-critical period-doubling bifurcation that occurs at amplitude a=0.08, as shown in Figure 20a. The newly established mixed state has a high degree of temporal order characterised by the sharp subharmonic and harmonic peaks in the frequency spectrum. At around a=0.15, the mixed state undergoes a secondary bifurcation that corresponds to the modulation instability of wave amplitude. While from the dynamical theory point of view, this instability corresponds to a torus bifurcation (tr), the resulting spectrum develops a number of equidistant frequency peaks that can be used as an AFC.

A qualitatively different scenario was obtained for f=28 Hz, see Figure 20b, where the primary instability of harmonic waves is most likely a torus bifurcation. When *a* is increased with step Δ, harmonic waves follow the branch shown by asterisks. They lose stability at around a=0.092 (labels (sn) and (tr)). The response amplitude jumps sharply and no subharmonic peak is visible in the temporal spectrum, thereby speaking in favour of a torus or saddle-node bifurcation. A new state corresponds to the modulated Faraday waves, which is confirmed by the presence of frequency sidebands in the temporal spectrum. These are required for the AFC generation. Moreover, as the vibration amplitude *a* is decreased from a=0.12, the response amplitude follows the branch shown by circles that stretches to a=0.07. At a=0.087 (label (tr)), the modulated Faraday waves are replaced with non-modulated standing waves via a reversed torus bifurcation. Yet, for a<0.07 (label (sn)) the branch of standing non-modulated Faraday waves does not exist and the drop response is harmonic. The subcriticality of bifurcations results in a hysteresis loop shown by the thick blue arrows in Figure 20b.

The physical mechanism behind the formation of the AFC in the regime of the modulational instability is the nonlinear mixing of the three frequencies: the driving frequency *f*, the sub-harmonic frequency of the Faraday waves f/2 and the much smaller frequency $f_{m}\ll f$, which corresponds to the amplitude modulation. A close-up of the temporal spectrum (3) from Figure 20b in the regime of the developed modulational instability is shown in Figure 20c. The dashed vertical lines highlight the location of the delta peaks in the power spectrum of the response of the drop. The peaks appear as equidistant, with a scaled inter-peak interval of $f/f_{0}=\Delta =0.04$, where $f_{0}=28$ Hz is the driving frequency.

**Figure 21:**
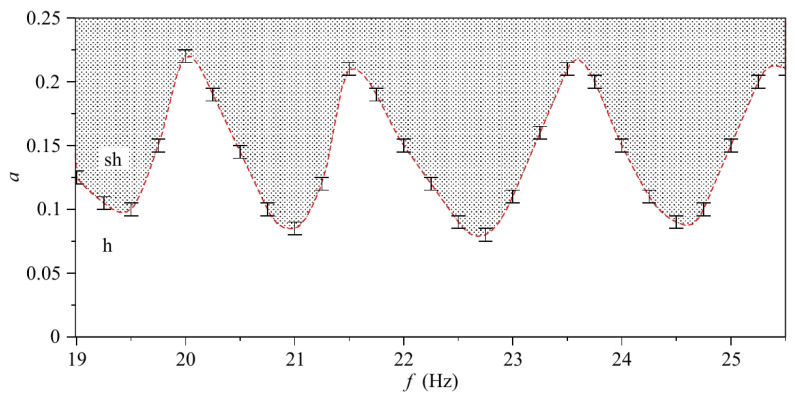
Frequency-amplitude phase diagram of the harmonic (h) and subharmonic (sh) responses for a fixed volume ethanol drop vertically vibrated with the frequency *f*. The response is subharmonic in the shaded area, where the generation of AFCs has been observed. Reproduced from [23]. Copyright 2019 by the American Physical Society.

As follows from the discussion (Figure 20), the AFC generation is possible only when the drop size and the frequency of its vertical vibrations fall within specific ranges. This observation was confirmed experimentally as shown in Figure 19, although slightly higher vibration frequencies were used. To visualise the existence range of AFCs, in Figure 21 we plot the boundary between harmonic and subharmonic responses in the frequency–amplitude plane for a fixed volume ethanol drop. The boundary is obtained by gradually increasing the vibration amplitude *a* at a fixed frequency *f*. The response is subharmonic in the shaded area, where the AFC generation has been observed. A high sensitivity of the Faraday instability threshold to frequency variation is explained by a simple geometric commensurability condition: if the horizontal drop size is an integer multiple of a half of the Faraday wavelength $\lambda_{F}/2=\pi$, then subharmonic Faraday waves are easier to excite on the drop surface.

Finally, note that both theory and experiment in [23] demonstrate that in highly viscous fluids the modulation instability of subharmonic Faraday waves is pushed towards larger values of vibration amplitude, thereby affecting the AFC existence conditions outlined above since in this case the primary instability of harmonic waves is dominated by a period-doubling bifurcation and modulation sidebands are not found in the frequency spectrum. Hence, we conclude that highly viscous liquids would be less suitable for the generation of Faraday wave-based AFCs.

### 7.5. Faraday Wave Liquid–Metal Drops at Room Temperature

In this section, we discuss the perspectives of the AFC generation using nonlinear acoustic properties of novel non-toxic, room-temperature liquid–metal materials notable for their unusual electronic, fluid mechanical and optical properties [25,26,27,192,193]. For example, in [27], eutectic gallium-indium alloy (EGaIn, 75% Ga 25% In by weight, melting point approximately $-15.5\;{}^{\circ }C$) spherical nanoparticles suspended in ethanol were investigated and their strong plasmonic resonances in the UV spectral range were demonstrated. Previously, nanoparticle-like liquid droplets were investigated as ultra-small mechanical resonators that can be used to generate FC-like signals [166,167]. However, liquids used in those studies did not support any plasmon mode because they did not possess the conductivity of metals. On the contrary, liquid metals combine the properties of both liquids and metals. Therefore, their drops can oscillate like pure liquid and exhibit plasmonic properties at the same time. This was demonstrated in [182]. Liquid metals also change their shape easily. For example, it was suggested in [193] that an acoustically driven oscillating gas bubble located above a liquid–metal layer can modify the liquid–metal surface, creating a parabolic-like micro-mirror that can focus UV light into a beam.

Here, we discuss another opportunity that liquid metals offer in the field of AFC generation using surface Faraday waves. EGaIn and similar Ga-based alloys have the following relevant material parameters: surface tension σ=624 mN/m, dynamic viscosity $\mu =1.99\;\times \;10^{-3}$ Pa s and density ρ=6280 kg/m^3^, which we use in calculations supporting this discussion. However, the surface of EGaIn is covered by a nanometer-thin oxide layer [25,27] that does not dissolve into the bulk metal and is also technologically important for the formation of stable drops. Furthermore, the surface tension of EGaIn is reversibly changeable from 0.624 N/m to 0.07 N/m (approximately the surface tension of water) when an external voltage is applied to liquid metal layers [25,26]. The use of these properties creates new opportunities for studies of Faraday waves and relevant nonlinear phenomena in liquid metals.

For example, the onset of Faraday waves on the surface of a large liquid pancake-like drop made of pure Ga was observed in an experiment, where the frequency of vertical vibration was varied from 40 to 300 Hz. It was found that the conditions for the generation of FC-like signals could be satisfied at much lower vibration amplitudes than those in the experiments involving ethanol and canola oil drops of similar dimensions [23]. These observations are in good agreement with the result of the analysis that we discuss below.

Note that the thickness *h* of the pancake-like drop of the liquid metal created on the surface of a vertically vibrated plate is not a free parameter but is related to the contact angle α as

(25) $$\left(1/2\right)\rho gh^{2}=\sigma \left[1-\cos\left(\alpha \right)\right]\;.$$

**Figure 22:**
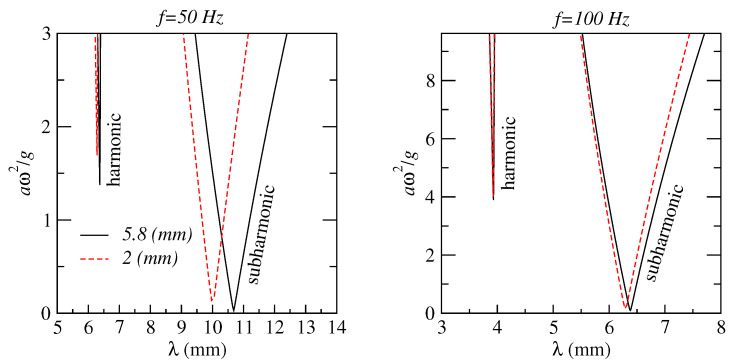
Stability diagram for h=5.6 mm and h=2 mm deep liquid metal layers showing two subharmonic Faraday tongues. The oscillation frequency of Faraday waves is half of the vertical vibration frequency f=50 Hz (**left**) and 100 Hz (**right**).

For example, using a typical value $\alpha =160^{\circ }$ [194], one obtains h=5.8 mm. Figure 22 shows the stability diagram for a liquid metal that demonstrates that the Faraday threshold for this material is much lower than that of ethanol in Figure 18a. Furthermore, this diagram indicates that at higher amplitudes *a* the excitation of harmonic perturbations could be observed, which further increases the magnitude of multiple spectrum peaks thus making them usable for AFC applications. An intuitive physical reason for a low Faraday threshold for a liquid metal is that the threshold is controlled by the kinematic viscosity η=μ/ρ, which is estimated as $1.5\;\times \;10^{-6}$ for ethanol and $3.7\;\times \;10^{-7}$ for liquid metal.

Of course, the use of Faraday waves on the surface of a liquid metal drop for AFC purposes has physical limitations. For example, it is technically challenging to excite sub-micrometer patterns that, for example, would be required to enable a strong interaction of light with the surface Faraday waves—such a wave pattern would be perceived by the light as an oscillating diffraction grating, which in turn could lead to intriguing optical effects in the frequency range, where liquid metal supports plasmon resonances [27]. In fact, the Faraday instability originates from a parametric excitation of gravity-capillary waves on the surface of a fluid. When the effect of viscosity is neglected, which is a valid approximation for liquid metals, the dispersion relation for gravity-capillary waves in shallow layers can be written as

(26) $$\omega^{2}=\left(gk+\sigma k^{3}/\rho \right)\tanh\left(kh\right)\;,$$

where ω is the driving angular frequency, *h* is the thickness of the layer and k=2π/λ is the Faraday wavenumber. When ω≫1, the wavenumber is also large so that the gravity in the dispersion relation can be neglected. This leads to the wavelength

(27) $$\lambda =2\pi {\left(\sigma /\left(\omega^{2}\rho \right)\right)}^{1/3}\;.$$

If ω=1 MHz then λ=14 μm. To excite a sub-micrometer wavelength, which would give rise to optical diffraction grating-like oscillations on the liquid metal surface, the frequency should be of order of several GHz. This presents a significant technical challenge because it is difficult to produce a mechanical vibration with the frequency in the GHz range. However, as discussed above, in liquid metal drops, Faraday waves could also be excited using an electrowetting technique [181,186]. As theoretically shown in [182], using electrowetting, one can excite oscillations of the liquid metal surface with GHz-range frequencies. Although the application of such results in the context of AFC generation was not the focus of the work [182], a dramatic change in the frequency of the plasmon resonance due to capillary oscillations found there unambiguously speaks in favour of the plausibility of optical wave modulation and the appearance of sideband peaks in the optical spectrum of the incident light, thus resulting in a signal that could be used as an AFC.

Thus, the application of non-toxic, room-temperature liquid–metal alloys holds the promise to become a useful method of AFC generation. While in many applications the use of solid-state AFCs could be preferred, in some situations liquid-state technologies can be advantageous. For example, liquid metals discussed in this section have the potential to be used inside a living body to realise such important functions as sensing and drug delivery [27,28,195]. Therefore, it is plausible that a combination of these new approaches with an FC-based medical technology [196] could become a subject of future research.

## 8. Conclusions and Outlook

There has been significant progress in understanding the fundamental physical processes that underpin the operation of AFCs—non-optical counterparts of OFCs. As with OFCs that are essential in applications that require the highest accuracy and resolution when measuring the frequency, time, distance or molecular composition of a material using light, AFCs have enabled similar functionalities using sound and vibrations in practical situations where light cannot be used. Similar to OFCs that have evolved into an independent technology soon after their introduction, the recently proposed AFCs have already enabled several novel spectroscopy and microscopy techniques, sensors and medical imaging modalities. Hence, it is plausible that the field of AFCs will rapidly grow and that this emergent technology will find further applications in science and engineering.

However, extra research and development work is required for the AFC technology to fulfil its full potential. Here, we outlined some of the important milestones that will need to be reached to achieve this goal.

Firstly, whereas AFCs are expected to operate similarly to OFCs, there are several differences between these two techniques, stemming from the disparate frequencies of acoustic waves (as well as the vibrations and spin waves) and light and also with physical mechanisms by which these waves interact with media. This aspect presents numerous technological challenges that shape research efforts in the field of AFCs.

Secondly, the type of AFC suitable for a particular practical application, and thus the physical mechanism of its generation, depends on specific experimental conditions and technical requirements. While this observation also applies to OFCs since they can be generated using a number of different techniques, in the case of AFCs, one has a much wider choice of approaches to the comb generation. For example, whereas generating AFC spectra with peaks of the same magnitude would be advantageous for certain applications, having peaks of different heights—which is the case with several AFCs discussed in this article—can be, in general, inconsequential as long as the peaks are detectable and their frequencies are stable.

Furthermore, some AFCs have a smaller number of spectral peaks than OFCs, which is a feature that has been identified as being important for a number of practical applications including phonon lasers [65,66] and computing [67]. Such AFCs should be compared with opto-electronic OFCs that are known to also have a small number of peaks and require special techniques for broadening their spectral ranges [137]. The same applies, although to a lesser degree, to Kerr OFCs generated using a cascade of optical FWM processes [33]. Indeed, a Kerr OFC may have just ten or so peaks due to the intrinsically low strength of nonlinear optical effects [8,41], which is in stark contrast to conventional OFCs based on mode-locked lasers and containing hundreds of frequency peaks [1].

Thirdly, although considerable attention has been paid to the long-term temporal stability of certain kinds of AFCs and conclusions have been drawn regarding their applicability to resolve specific problems such as precision underwater measurement, the stability of AFCs of the other kinds has not been investigated in great detail. While filling this knowledge gap is likely to be one of the future directions in AFC research, the lack of long-term stability may be inconsequential in some applications such as, for example, sensing and imaging in in vivo biological environments [7,183] that evolve in time.

To conclude, due to the aforementioned differences between acoustic and optical frequency combs, so far they have been mostly targeted at different technological applications. For this reason, a direct quantitative comparison of their performance at present may be difficult or even impossible. However, the field of AFCs is developing rapidly and catching up with its more mature OFC counterpart. For example, OFCs have been used to control quantum bits (qubits) for more than a decade [197], but very recently the possibility of using AFCs for a qubit manipulation has also been demonstrated [198]. Another example of applications where AFCs start competing with OFCs is precision measurements in Bose–Einstein condensate systems [51]. Moreover, both OFCs and AFCs can potentially find applications in enhancing the sensitivity of resonant-mode sensors or for improving the phase noise of oscillators or phase locked systems [51]. Thus, whereas the development pathway of AFCs is different from that of OFCs, the former have already reached the patent stage (see, e.g., [199,200]). This means that they are very close to entering scientific and commercial service as capable and versatile competitors to more traditional OFC systems.

## Abbreviations

The following abbreviations are used in this manuscript:

AFC	Acoustic frequency comb
APD	Avalanche photodiode
BAW	Bulk acoustic wave
BLS	Brillouin light scattering
BS	Beam splitter
CW	Continuous wave
EGaIn	Eutectic gallium-indium alloy
FC	Frequency comb
FWM	Four wave mixing
LDV	Laser Doppler vibrometer
MOKE	Magneto-optical Kerr effect
OFC	Optical frequency comb
PBS	Polarising beam splitter
PSD	Power spectral density
SAW	Surface acoustic wave
SBS	Stimulated Brillouin scattering
SERS	Surface-enhanced Raman scattering
SONAR	Sound navigation and ranging
SW	Spin wave
TFPI	Tandem Fabry–Pérot interferometer
TLS	Two level system
UV	Ultraviolet
VIPA	Virtual image phase array
VP	Velocity profiler
WGM	Whispering gallery mode

## References

[1] Picqué N., Hänsch T.W. (2019). Frequency comb spectroscopy. Nat. Photonics.

[2] Weichman M.L., Changala P.B., Ye J., Chen Z., Yan M., Picqué N. (2019). Broadband molecular spectroscopy with optical frequency combs. J. Mol. Spectrosc..

[3] Fortier T., Baumann E. (2019). 20 years of developments in optical frequency comb technology and applications. Commun. Phys..

[4] Urick R.J. (1983). Principles of Underwater Sound.

[5] Wu H., Qian Z., Zhang H., Xu X., Xue B., Zhai J. (2019). Precise underwater distance measurement by dual acoustic frequency combs. Ann. Phys..

[6] Wang B., Su J.L., Karpiouk A.B., Sokolov K.V., Smalling R.W., Emelianov S.Y. (2010). Intravascular photoacoustic imaging. IEEE J. Sel. Top. Quantum Electron..

[7] Maksymov I.S. (2016). Magneto-plasmonic nanoantennas: Basics and applications. Rev. Phys..

[8] Maksymov I.S., Greentree A.D. (2019). Coupling light and sound: Giant nonlinearities from oscillating bubbles and droplets. Nanophotonics.

[9] Ganesan A., Do C., Seshia A. (2017). Phononic frequency comb via intrinsic three-wave mixing. Phys. Rev. Lett..

[10] Eggleton B.J., Poulton C.G., Rakich P.T., Steel M.J., Bahl G. (2019). Brillouin integrated photonics. Nat. Photonics.

[11] Laude V., Beugnot J.C., Sylvestre T. (2019). Special issue on Brillouin scattering and optomechanics. Appl. Sci..

[12] Kang M.S., Butsch A., Russell P.S.J. (2011). Reconfigurable light-driven opto-acoustic isolators in photonic crystal fibre. Nat. Photonics.

[13] Shen Z., Zhang Y.L., Chen Y., Sun F.W., Zou X.B., Guo G.C., Zou C.L., Dong C.H. (2018). Reconfigurable optomechanical circulator and directional amplifier. Nat. Commun..

[14] Qi Z., Menyuk C.R., Gorman J.J., Ganesan A. (2020). Existence conditions for phononic frequency combs. Appl. Phys. Lett..

[15] Goryachev M., Galliou S., Tobar M.E. (2020). Generation of ultralow power phononic combs. Phys. Rev. Res..

[16] Meng Z., Yakovlev V.V., Utegulov Z. Surface-enhanced Brillouin scattering in a vicinity of plasmonic gold nanostructures. Proceedings of the Plasmonics in Biology and Medicine XII.

[17] Bai Z., Yuan H., Liu Z., Xu P., Gao Q., Williams R.J., Kitzler O., Mildren R.P., Wang Y., Lu Z. (2018). Stimulated Brillouin scattering materials, experimental design and applications: A review. Opt. Mater..

[18] Muralidhar S., Awad A.A., Alemán A., Khymyn R., Dvornik M., Hanstorp D., Åkerman J. (2020). Sustained coherent spin wave emission using frequency combs. Phys. Rev. B.

[19] Maksymov I.S., Greentree A.D. (2016). Plasmonic nanoantenna hydrophones. Sci. Rep..

[20] Maksymov I.S., Greentree A.D. (2017). Synthesis of discrete phase-coherent optical spectra from nonlinear ultrasound. Opt. Express.

[21] Nguyen B.Q.H., Maksymov I.S., Suslov S.A. (2021). Acoustic frequency combs using gas bubble cluster oscillations in liquids: A proof of concept. Sci. Rep..

[22] Nguyen B.Q.H., Maksymov I.S., Suslov S.A. (2021). Spectrally wide acoustic frequency combs generated using oscillations of polydisperse gas bubble clusters in liquids. Phys. Rev. E.

[23] Maksymov I.S., Pototsky A. (2019). Harmonic and subharmonic waves on the surface of a vibrated liquid drop. Phys. Rev. E.

[24] Maksymov I.S., Pototsky A., Greentree A.D. Optical frequency comb by giant nonlinear capillary waves. Proceedings of the SPIE Micro + Nano Materials, Devices, and Applications 2019.

[25] Dickey M.D. (2017). Stretchable and Soft Electronics using Liquid Metals. Adv. Mater..

[26] Daeneke T., Khoshmanesh K., Mahmood N., Castro I.A.D., Esrafilzadeh D., Barrow S., Dickey M., Zadeh K.K. (2018). Liquid metals: Fundamentals and applications in chemistry. Chem. Soc. Rev..

[27] Reineck P., Lin Y., Gibson B.C., Greentree A.D., Maksymov I.S. (2019). UV plasmonic properties of colloidal liquid–metal eutectic gallium-indium alloy nanoparticles. Sci. Rep..

[28] Kalantar-Zadeh K., Tang J., Daeneke T., O’Mullane A.P., Stewart L.A., Liu J., Majidi C., Ruoff R.S., Weiss P.S., Dickey M.D. (2019). Emergence of liquid metals in nanotechnology. ACS Nano.

[29] Hall J. (2000). Optical frequency measurement: 40 years of technology revolutions. IEEE J. Sel. Top. Quantum Electron..

[30] Hänsch T.W. (2006). Nobel lecture: Passion for precision. Rev. Mod. Phys..

[31] Hall J.L. (2006). Nobel lecture: Defining and measuring optical frequencies. Rev. Mod. Phys..

[32] Cundiff S.T., Ye J., Hall J.L. (2001). Optical frequency synthesis based on mode-locked lasers. Rev. Sci. Instrum..

[33] Chembo Y.K. (2016). Kerr optical frequency combs: Theory, applications and perspectives. Nanophotonics.

[34] Wilken T., Lo Curto R.A.P.G., Steinmetz T., Manescau A., Pasquini L., Hernández J.I.G., Rebolo R., Hänsch T.W., Udem T., Holzwarth R. (2012). A spectrograph for exoplanet observations calibrated at the centimetre-per-second level. Nature.

[35] Baltuška A., Udem T., Uiberacker M., Hentschel M., Goulielmakis E., Gohle C., Holzwarth R., Yakovlev V.S., Scrinzi A., Hänsch T.W. (2003). Attosecond control of electronic processes by intense light fields. Nature.

[36] Torres-Company V., Weiner A.M. (2015). Optical frequency comb technology for ultra-broadband radio-frequency photonics. Anal. Chem..

[37] Ye J., Cundiff S.T. (2005). Femtosecond Optical Frequency Comb: Principle, Operation and Applications.

[38] Maddaloni P., Bellini M., de Natale P. (2013). Laser-Based Measurements for Time and Frequency Domain Applications: A Handbook.

[39] Boyd R.W. (2008). Nonlinear Optics.

[40] Sefler G.A., Kitayama K. (1998). Frequency comb generation by four-wave mixing and the role of fiber dispersion. J. Lightwave Technol..

[41] Maksymov I.S., Miroshnichenko A.E., Kivshar Y.S. (2013). Cascaded four-wave mixing in tapered plasmonic nanoantenna. Opt. Lett..

[42] Wu J., Xu X., Nguyen T.G., Chu S.T., Little B.E., Morandotti R., Mitchell A., Moss D.J. (2018). RF photonics: An optical microcombs’ perspective. IEEE J. Sel. Top. Quantum Electron..

[43] Pasquazi A., Peccianti M., Razzari L., Mossc D.J., Coen S., Erkintalo M., Chembo Y.K., Hansson T., Wabnitz S., Del’Haye P. (2018). Micro-combs: A novel generation of optical sources. Phys. Rep..

[44] Faist J., Villares G., Scalari G., Rösch M., Bonzon C., Hugi A., Beck M. (2016). Quantum cascade laser frequency combs. Nanophotonics.

[45] Murata H., Morimoto A., Kobayashi T., Yamamoto S. (2000). Optical pulse generation by electrooptic-modulation method and its application to integrated ultrashort pulse generators. IEEE J. Sel. Top. Quantum Electron..

[46] Mackintosh A.N., Anderson B.M., Lorrey A.M., Renwick J.A., Frei P., Dean S.M. (2017). Regional cooling caused recent New Zealand glacier advances in a period of global warming. Nat. Commun..

[47] Cao L.S., Qi D.X., Peng R.W., Wang M., Schmelcher P. (2014). Phononic frequency combs through nonlinear resonances. Phys. Rev. Lett..

[48] Xiong H., Si L.G., Lü X.Y., Wu Y. (2016). Optomechanically induced sum sideband generation. Opt. Express.

[49] Cao C., Mi S.C., Wang T.J., Zhang R., Wang C. (2016). Optical high-order sideband comb generation in a photonic molecule optomechanical system. IEEE J. Quantum Electron..

[50] Ganesan A., Seshia A. (2019). Resonance tracking in a micromechanical device using phononic frequency combs. Sci. Rep..

[51] Kubena R.L., Wall W.S., Koehl J., Joyce R.J. (2020). Phononic comb generation in high-Q quartz resonators. Appl. Phys. Lett..

[52] Mercadé L., Martín L.L., Griol A., Navarro-Urrios D., Martínez A. (2020). Microwave oscillator and frequency comb in a silicon optomechanical cavity with a full phononic bandgap. Nanophotonics.

[53] Rudenko O.V. (2006). Giant nonlinearities in structurally inhomogeneous media and the fundamentals of nonlinear acoustic diagnostic technique. Phys. Usp..

[54] Fabelinskii I.L. (1969). Molecular Scattering of Light.

[55] Garmire E. (2018). Stimulated Brillouin review: Invented 50 years ago and applied today. Int. J. Opt..

[56] Traverso A.J., Thompson J.V., Steelman Z.A., Meng Z., Scully M.O., Yakovlev V.V. (2013). Dual Raman-Brillouin microscope for chemical and mechanical characterization and imaging. Laser Photonics Rev..

[57] Ballmann C.W., Thompson J.V., Traverso A.J., Meng Z., Scully M.O., Yakovlev V.V. (2015). Stimulated Brillouin scattering microscopic imaging. Sci. Rep..

[58] Meng Z., Traverso A.J., Ballmann C.W., Troyanova-Wood M.A., Yakovlev V.V. (2016). Seeing cells in a new light: A renaissance of Brillouin spectroscopy. Adv. Opt. Photonics.

[59] Palombo F., Fioretto D. (2019). Brillouin light scattering: Applications in biomedical sciences. Chem. Rev..

[60] Remer I., Shaashoua R., Shemesh N., Ben-Zvi A., Bilenca A. (2020). High-sensitivity and high-specificity biomechanical imaging by stimulated Brillouin scattering microscopy. Nat. Methods.

[61] Sader J.E., Chon J.W.M., Mulvaney P. (1999). Calibration of rectangular atomic force microscope cantilevers. Rev. Sci. Instrum..

[62] Onorato M., Vozella L., Proment D., Lvov Y.V. (2015). Route to thermalization in the *α*-Fermi-Pasta-Ulam system. Proc. Natl. Acad. Sci. USA.

[63] Rabinovich M.I., Trubetskov D.I. (1989). Oscillations and Waves in Linear and Nonlinear Systems.

[64] Kumazawa M., Higashihara H., Nagai T. Development of acoustic frequency comb technology by ACROSS appropriate for active monitoring of the earthquake field. Proceedings of the Japan Geoscience Union Meeting.

[65] Grudinin I.S., Lee H., Painter O., Vahala K.J. (2010). Phonon laser action in a tunable two-level system. Phys. Rev. Lett..

[66] Beardsley R.P., Akimov A.V., Henini M., Kent A.J. (2010). Coherent terahertz sound amplification and spectral line narrowing in a Stark ladder superlattice. Phys. Rev. Lett..

[67] Stannigel K., Komar P., Habraken S.J.M., Bennett S.D., Lukin M.D., Zoller P., Rabl P. (2012). Optomechanical quantum information processing with photons and phonons. Phys. Rev. Lett..

[68] Maksymov I.S. (2018). Perspective: Strong microwave photon-magnon coupling in multiresonant dielectric antennas. J. Appl. Phys..

[69] Baity P.G., Bozhko D.A., Macědo R., Smith W., Holland R.C., Danilin S., Seferai V., Barbosa J., Peroor R.R., Goldman S. (2021). Strong magnon–photon coupling with chip-integrated YIG in the zero-temperature limit. Appl. Phys. Lett..

[70] Nagel M., Parker S.R., Kovalchuk E.V., Stanwix P.L., Hartnett J.G., Ivanov E.N., Peters A., Tobar M.E. (2015). Direct terrestrial test of Lorentz symmetry in electrodynamics to 10^−18^. Nat. Commun..

[71] Lo A., Haslinger P., Mizrachi E., Anderegg L., Múller H., Hohensee M., Goryachev M., Tobar M.E. (2016). Acoustic tests of Lorentz symmetry using quartz oscillators. Phys. Rev. X.

[72] Goryachev M., Kuang Z., Ivanov E.N., Haslinger P., Müller H., Tobar M.E. (2018). Next generation of phonon tests of Lorentz invariance using quartz BAW resonators. IEEE Trans. Ultrason. Ferroelectr. Freq. Control.

[73] Shao C.G., Chen Y.F., Tan Y.J., Yang S.Q., Luo J., Tobar M.E., Long J.C., Weisman E., Kostelecký V.A. (2019). Combined Search for a Lorentz-Violating Force in Short-Range Gravity Varying as the Inverse Sixth Power of Distance. Phys. Rev. Lett..

[74] Guéna J., Abgrall M., Rovera D., Rosenbusch P., Tobar M.E., Laurent P., Clairon A., Bize S. (2012). Improved tests of local position invariance using ^87^Rb and ^133^Cs fountains. Phys. Rev. Lett..

[75] Goryachev M., McAllister B.T., Tobar M.E. (2019). Axion detection with precision frequency metrology. Phys. Dark Universe.

[76] Johannsmann D. (2010). Viscoelastic, mechanical, and dielectric measurements on complex samples with the quartz crystal microbalance. Phys. Chem. Chem. Phys..

[77] Roque T.F., Marquardt F., Yevtushenko O.M. (2020). Nonlinear dynamics of weakly dissipative optomechanical systems. New J. Phys..

[78] Galliou S., Goryachev M., Bourquin R., Abbé P., Aubry J.P., Tobar M.E. (2013). Extremely low loss phonon-trapping cryogenic acoustic cavities for future physical experiments. Sci. Rep..

[79] Nosek J. (1999). Drive level dependence of the resonant frequency in BAW quartz resonators and his modeling. IEEE Trans. Ultrason. Ferroelectr. Freq. Control.

[80] Lisenfeld J., Grabovskij G.J., Müller C., Cole J.H., Weiss G., Ustinov A.V. (2015). Observation of directly interacting coherent two-level systems in an amorphous material. Nat. Commun..

[81] Poddubny A.N., Poshakinskiy A.V., Jusserand B., Lemaître A. (2014). Resonant Brillouin scattering of excitonic polaritons in multiple-quantum-well structures. Phys. Rev. B.

[82] Maksymov I.S., Kostylev M. (2015). Broadband stripline ferromagnetic resonance spectroscopy of ferromagnetic films, multilayers and nanostructures. Physica E.

[83] Demokritov S.O., Hillebrands B., Slavin A.N. (2001). Brillouin light scattering studies of confined spin waves: Linear and nonlinear confinement. Phys. Rep..

[84] Stashkevich A.A., Djemia P., Fetisov Y.K., Bizière N., Fermon C. (2007). High-intensity Brillouin light scattering by spin waves in a permalloy film under microwave resonance pumping. J. Appl. Phys..

[85] Gubbiotti G., Tacchi S., Madami M., Carlotti G., Adeyeye A.O., Kostylev M. (2010). Brillouin light scattering studies of planar metallic magnonic crystals. J. Phys. D Appl. Phys..

[86] Serga A.A., Sandweg C.W., Vasyuchka V.I., Jungfleisch M.B., Hillebrands B., Kreisel A., Kopietz P., Kostylev M.P. (2012). Brillouin light scattering spectroscopy of parametrically excited dipole-exchange magnons. Phys. Rev. B.

[87] Sebastian T., Schultheiss K., Obry B., Hillebrands B., Schultheiss H. (2015). Micro-focused Brillouin light scattering: Imaging spin waves at the nanoscale. Front. Phys..

[88] Maksymov I.S. (2015). Magneto-plasmonics and resonant interaction of light with dynamic magnetisation in metallic and all-magneto-dielectric nanostructures. Nanomaterials.

[89] Akilbekova D., Ogay V., Yakupov T., Sarsenova M., Umbayev B., Nurakhmetov A., Tazhin K., Yakovlev V.V., Utegulov Z.N. (2018). Brillouin spectroscopy and radiography for assessment of viscoelastic and regenerative properties of mammalian bones. J. Biomed. Opt..

[90] Garmire E., Townes C.H. (1964). Stimulated Brillouin scattering in liquids. Appl. Phys. Lett..

[91] Braje D., Hollberg L., Diddams S. (2009). Brillouin-enhanced hyperparametric generation of an optical frequency comb in a monolithic highly nonlinear fiber cavity pumped by a cw laser. Phys. Rev. Lett..

[92] Lin G., Diallo S., Saleh K., Martinenghi R., Beugnot J.C., Sylvestre T., Chembo Y.K. (2014). Cascaded Brillouin lasing in monolithic barium fluoride whispering gallery mode resonators. Appl. Phys. Lett..

[93] Lu Q., Liu S., Wu X., Liu L., Xu L. (2016). Stimulated Brillouin laser and frequency comb generation in high-Q microbubble resonators. Opt. Lett..

[94] Dong M., Winful H.G. (2016). Unified approach to cascaded stimulated Brillouin scattering and frequency-comb generation. Phys. Rev. A.

[95] Mock R., Hillebrands B., Sandercock R. (1987). Construction and performance of a Brillouin scattering set-up using a triple-pass tandem Fabry–Pérot interferometer. J. Phys. E Sci. Instrum..

[96] Scarcelli G., Yun S.H. (2008). Confocal Brillouin microscopy for three-dimensional mechanical imaging. Nat. Photonics.

[97] Alemán A., Muralidhar S., Awad A.A., Åkerman J., Hanstorp D. (2020). Frequency comb enhanced Brillouin microscopy. Opt. Express.

[98] Raether H. (1987). Surface Plasmons on Smooth and Rough Surfaces and on Gratings.

[99] Enoch S., Bonod N. (2012). Plasmonics: From Basic to Advanced Topics.

[100] Kauranen M., Zayats A.V. (2012). Nonlinear plasmonics. Nat. Photonics.

[101] Panoiu N.C., Sha W.E.I., Lei D.Y., Li G.C. (2018). Nonlinear optics in plasmonic nanostructures. J. Opt..

[102] Mayer K.M., Hafner J.H. (2011). Localized surface plasmon resonance sensors. Chem. Rev..

[103] Kostylev N., Maksymov I.S., Adeyeye A.O., Samarin S., Kostylev M., Williams J.F. (2013). Plasmon-assisted high reflectivity and strong magneto-optical Kerr effect in permalloy gratings. Appl. Phys. Lett..

[104] Zvezdin A.K., Kotov V.A. (1997). Modern Magnetooptics and Magnetooptical Materials.

[105] Bonanni V., Bonetti S., Pakizeh T., Pirzadeh Z., Chen J., Nogués J., Vavassori P., Hillenbrand R., Åkerman J., Dmitriev A. (2011). Designer magnetoplasmonics with nickel nanoferromagnets. Nano Lett..

[106] Chen J., Albella P., Pirzadeh Z., Alonso-González P., Huth F., Bonetti S., Bonanni V., Åkerman J., Nogués J., Vavassori P. (2011). Plasmonic nickel nanoantennas. Small.

[107] Chetvertukhin A.V., Baryshev A.V., Uchida H., Inoue M., Fedyanin A.A. (2012). Resonant surface magnetoplasmons in two-dimensional magnetoplasmonic crystals excited in Faraday configuration. J. Appl. Phys..

[108] Temnov V.V. (2012). Ultrafast acousto-magneto-plasmonics. Nat. Photonics.

[109] Armelles G., Cebollada A., García-Martín A., González M.U. (2013). Magnetoplasmonics: Combining magnetic and plasmonic functionalities. Adv. Opt. Mater..

[110] Chin J.Y., Steinle T., Wehlus T., Dregely D., Weiss T., Belotelov V.I., Stritzker B., Giessen H. (2013). Nonreciprocal plasmonics enables giant enhancement of thin-film Faraday rotation. Nat. Photonics.

[111] Li J., Zhang G., Wang J., Maksymov I.S., Greentree A.D., Hu J., Shen A., Wang Y., Trau M. (2018). Facile one-pot synthesis of nanodot-decorated gold-silver alloy nanoboxes for single-particle surface-enhanced Raman scattering activity. ACS Appl. Mater. Interfaces.

[112] Li D., Jiang L., Piper J.A., Maksymov I.S., Greentree A.D., Wang E., Wang Y. (2019). Sensitive and multiplexed SERS nanotags for the detection of cytokines secreted by lymphoma. ACS Sens..

[113] Thyagarajan K., Rivier S., Lovera A., Martin O.J.F. (2012). Enhanced second-harmonic generation from double resonant plasmonic antennae. Opt. Express.

[114] Aouani H., Rahmani M., Navarro-Cía M., Maier S.A. (2013). Third-harmonic-upconversion enhancement from a single semiconductor nanoparticle coupled to a plasmonic antenna. Nat. Nanotechnol..

[115] Palomba S., Novotny L. (2008). Nonlinear excitation of surface plasmon polaritons by four-wave mixing. Phys. Rev. Lett..

[116] Maksymov I.S., Greentree A.D. (2015). Detecting nonlinear acoustic waves in liquids with nonlinear dipole optical antennae. arXiv.

[117] Maksymov I.S., Greentree A.D. Plasmon-enhanced Brillouin light scattering from nonlinear acoustic waves. Proceedings of the 1st International Workshop on Optomechanics and Brillouin Scattering (WOMBAT).

[118] Beranek L.L. (1996). Acoustics.

[119] Salem R., Foster M.A., Turner A.C., Geraghty D.F., Lipson M., Gaeta A.L. (2008). Signal regeneration using low-power four-wave mixing on silicon chip. Nat. Photonics.

[120] Ferrera M., Razzari L., Duchesne D., Morandotti R., Yang Z., Liscidini M., Sipe J.E., Chu S., Little B.E., Moss D.J. (2008). Low-power continuous-wave nonlinear optics in doped silica glass integrated waveguide structures. Nat. Photonics.

[121] Li J., Lee H., Chen T., Vahala K.J. (2012). Low-pump-power, low-phase-noise, and microwave to millimeter-wave repetition rate operation in microcombs. Phys. Rev. Lett..

[122] Alam M.Z., Leon I.D., Boyd R.W. (2016). Large optical nonlinearity of indium tin oxide in its epsilon-near-zero region. Science.

[123] Rayleigh L. (1917). On the pressure developed in a liquid during the collapse of a spherical cavity. Philos. Mag..

[124] Minnaert M. (1933). On musical air-bubbles and the sound of running water. Philos. Mag..

[125] Plesset M.S. (1949). The dynamics of cavitation bubbles. J. Appl. Mech..

[126] Prosperetti A. (1974). Nonlinear oscillations of gas bubbles in liquids: Steady-state solutions. J. Acoust. Soc. Am..

[127] Francescutto A., Nabergoj R. (1983). Steady-state oscillations of gas bubbles in liquids: Explicit formulas for frequency response curves. J. Acoust. Soc. Am..

[128] Francescutto A., Nabergoj R. (1984). A multiscale analysis of gas bubble oscillations: Transient and steady-state solutions. Acoustica.

[129] Keller J.B., Miksis M. (1980). Bubble oscillations of large amplitude. J. Acoust. Soc. Am..

[130] Brennen C.E. (1995). Cavitation and Bubble Dynamics.

[131] Mettin R., Akhatov I., Parlitz U., Ohl C.D., Lauterborn W. (1997). Bjerknes forces between small cavitation bubbles in a strong acoustic field. Phys. Rev. E.

[132] Lauterborn W., Kurz T. (2010). Physics of bubble oscillations. Rep. Prog. Phys..

[133] Doinikov A.A. (2005). Bubble and Particle Dynamics in Acoustic Fields: Modern Trends and Applications.

[134] Suslov S.A., Ooi A., Manasseh R. (2012). Nonlinear dynamic behavior of microscopic bubbles near a rigid wall. Phys. Rev. E.

[135] Dzaharudin F., Suslov S.A., Manasseh R., Ooi A. (2013). Effects of coupling, bubble size, and spatial arrangement on chaotic dynamics of microbubble cluster in ultrasonic fields. J. Acoust. Soc. Am..

[136] Hwang P.A., Teague W.J. (2000). Low-Frequency Resonant Scattering of Bubble Clouds. J. Atmos. Ocean. Technol..

[137] Zhang M., Buscaino B., Wang C., Shams-Ansari A., Reimer C., Zhu R., Kahn J.M., Lončar M. (2019). Broadband electro-optic frequency comb generation in a lithium niobate microring resonator. Nature.

[138] Zabolotskaya A.E. (1984). Interaction of gas bubbles in a sound field. Sov. Phys. Acoust..

[139] Watanabe T., Kukita Y. (1993). Translational and radial motions of a bubble in an acoustic standing wave field. Phys. Fluids A.

[140] Pelekasis N.A., Tsamopuolos J.A. (1993). Bjerknes forces between two bubbles. Part 2. Response to an oscillatory pressure field. J. Fluid Mech..

[141] Doinikov A.A., Zavtrak S.T. (1995). On the mutual interaction of two gas bubbles in a sound field. Phys. Fluids.

[142] Barbat T., Ashgriz N., Liu C.S. (1999). Dynamics of two interacting bubbles in an acoustic field. J. Fluid Mech..

[143] Harkin A., Kaper T.J., Nadim A. (2001). Coupled pulsation and translation of two gas bubbles in a liquid. J. Fluid. Mech..

[144] Doinikov A.A. (2001). Translational motion of two interacting bubbles in a strong acoustic field. Phys. Rev. E.

[145] Macdonald C.A., Gomatam J. (2006). Chaotic dynamics of microbubbles in ultrasonic fields. Proc. Inst. Mech. Eng. Part C J. Mech. Eng. Sci..

[146] Mettin R., Doinikov A.A. (2009). Translational instability of a spherical bubble in a standing ultrasound wave. Appl. Acoust..

[147] Lanoy M., Derec C., Tourin A., Leroy V. (2015). Manipulating bubbles with secondary Bjerknes forces. Appl. Phys. Lett..

[148] Bjerknes V. (1906). Fields of Force.

[149] Leighton T.G., Walton A.J., Pickworth M.J.W. (1990). Primary Bjerknes forces. Eur. J. Phys..

[150] Kazantsev G.N. (1960). The motion of gaseous bubbles in a liquid under the influence of Bjerknes forces arising in an acoustic field. Sov. Phys. Dokl..

[151] Crum L.A. (1975). Bjerknes forces on bubbles in a stationary sound field. J. Acoust. Soc. Am..

[152] Doinikov A.A. (2004). Mathematical model for collective bubble dynamics in strong ultrasound fields. J. Acoust. Soc. Am..

[153] Chen S.H., Huang J.L., Sze K.Y. (2007). Multidimensional Lindstedt–Poincaré method for nonlinear vibration of axially moving beams. J. Sound Vib..

[154] Manasseh R., Ooi A. (2016). Frequencies of acoustically interacting bubbles. Bubble Sci. Eng. Technol..

[155] Tsamopoulos J.A., Brown R.A. (1983). Nonlinear oscillations of inviscid drops and bubbles. J. Fluid Mech..

[156] de Gennes P.G., Brochard-Wyart F., Quéré D. (2004). Capillarity and Wetting Phenomena: Drops, Bubbles, Pearls, Waves.

[157] Yoshiyasu N., Matsuda K., Takaki R. (1996). Self-induced vibration of a water drop placed on an oscillating plate. J. Phys. Soc. Jpn..

[158] John K., Thiele U. (2010). Self-ratcheting Stokes drops driven by oblique vibrations. Phys. Rev. Lett..

[159] Pucci G., Fort E., Ben Amar M., Couder Y. (2011). Mutual adaptation of a Faraday instability pattern with its flexible boundaries in floating fluid drops. Phys. Rev. Lett..

[160] Pucci G., Ben Amar M., Couder Y. (2013). Faraday instability in floating liquid lenses: The spontaneous mutual adaptation due to radiation pressure. J. Fluid Mech..

[161] Blamey J., Yeo L.Y., Friend J.R. (2013). Microscale capillary wave turbulence excited by high frequency vibration. Langmuir.

[162] Bostwick J.B., Steen P.H. (2014). Dynamics of sessile drops. Part 1. Inviscid theory. J. Fluid Mech..

[163] Chang C.T., Bostwick J.B., Daniel S., Steen P.H. (2015). Dynamics of sessile drops. Part 2. Experiment. J. Fluid Mech..

[164] Ebata H., Sano M. (2015). Swimming droplets driven by a surface wave. Sci. Rep..

[165] Hemmerle A., Froehlicher G., Bergeron V., Charitat T., Farago J. (2015). Worm-like instability of a vibrated sessile drop. EPL.

[166] Dahan R., Martin L.L., Carmon T. (2016). Droplet optomechanics. Optica.

[167] Maayani S., Martin L.L., Kaminski S., Carmon T. (2016). Cavity optocapillaries. Optica.

[168] Kaminski S., Martin L.L., Maayani S., Carmon T. (2016). Ripplon laser through stimulated emission mediated by water waves. Nat. Photonics.

[169] Childress L., Schmidt M.P., Kashkanova A.D., Brown C.D., Harris G.I., Aiello A., Marquardt F., Harris J.G.E. (2017). Cavity optomechanics in a levitated helium drop. Phys. Rev. A.

[170] Pototsky A., Bestehorn M. (2018). Shaping liquid drops by vibration. EPL (Europhys. Lett.).

[171] Valani R.N., Slim A.C., Simula T. (2019). Superwalking droplets. Phys. Rev. Lett..

[172] Benjamin T.B., Ursell F.J., Taylor G.I. (1954). The stability of the plane free surface of a liquid in vertical periodic motion. Proc. R. Soc. Lond. A.

[173] Henderson D.M., Miles J.W. (1990). Single-mode Faraday waves in small cylinders. J. Fluid Mech..

[174] Miles J.W. (1984). Nonlinear Faraday resonance. J. Fluid Mech..

[175] Jiang L., Ting C.L., Perlin M., Schultz W.W. (1996). Moderate and steep Faraday waves: Instabilities, modulation and temporal asymmetries. J. Fluid Mech..

[176] Shats M., Xia H., Punzmann H. (2012). Parametrically excited water surface ripples as ensembles of oscillons. Phys. Rev. Lett..

[177] Punzmann H., Shats M.G., Xia H. (2009). Phase randomization of three-wave interactions in capillary waves. Phys. Rev. Lett..

[178] Xia H., Maimbourg T., Punzmann H., Shats M. (2012). Oscillon dynamics and rogue wave generation in Faraday surface ripples. Phys. Rev. Lett..

[179] Turton S.E., Couchman M.M.P., Bush J.W.M. (2008). A review of the theoretical modeling of walking droplets: Toward a generalized pilotwave framework. Chaos.

[180] Tarasov N., Perego A.M., Churkin D.V., Staliunas K., Turitsyn S.K. (2016). Mode-locking via dissipative Faraday instability. Nat. Commun..

[181] Oh J.M., Ko S.H., Kang K.H. (2008). Shape oscillation of a drop in ac electrowetting. Langmuir.

[182] Maksymov I.S., Greentree A.D. (2017). Dynamically reconfigurable plasmon resonances enabled by capillary oscillations of liquid–metal nanodroplets. Phys. Rev. A.

[183] Maksymov I.S., Pototsky A. (2020). Excitation of Faraday-like body waves in vibrated living earthworms. Sci. Rep..

[184] Pototsky A., Maksymov I.S., Suslov S.A., Leontini J. (2020). Intermittent dynamic bursting in vertically vibrated liquid drops. Phys. Fluids.

[185] Pototsky A., Oron A., Bestehorn M. (2021). Equilibrium shapes and floatability of static and vertically vibrated heavy liquid drops on the surface of a lighter fluid. J. Fluid Mech..

[186] Tsai C.S., Mao R.W., Tsai S.C., Shahverdi K., Zhu Y., Lin S.K., Hsu Y.H., Boss G., Brenner M., Mahon S. (2017). Faraday waves-based integrated ultrasonic micro-droplet generator and applications. Micromachines.

[187] Shats M., Punzmann H., Xia H. (2010). Capillary rogue waves. Phys. Rev. Lett..

[188] Rajchenbach J., Leroux A., Clamond D. (2011). New standing solitary waves in water. Phys. Rev. Lett..

[189] Huh C., Scriven L.E. (1971). Hydrodynamic model of steady movement of a solid/liquid/fluid contact line. J. Colloid Interface Sci..

[190] Schwartz L.W., Eley R.R. (1998). Simulation of droplet motion on low-energy and heterogeneous surfaces. J. Colloid Interface Sci..

[191] Bestehorn M., Pototsky A. (2016). Faraday instability and nonlinear pattern formation of a two-layer system: A reduced model. Phys. Rev. Fluids.

[192] Neumann T.V., Dickey M.D. (2020). Liquid metal direct write and 3D printing: A review. Adv. Mater. Technol..

[193] Boyd B., Suslov S.A., Becker S., Greentree A.D., Maksymov I.S. (2020). Beamed UV sonoluminescence by aspherical air bubble collapse near liquid–metal microparticles. Sci. Rep..

[194] Liu T., Sen P., Kim C.J. (2012). Characterization of nontoxic liquid–metal alloy Galinstan for applications in microdevices. J. Microelectromech. Syst.

[195] Lu Y., Hu Q., Lin Y., Pacardo D.B., Wang C., Sun W., Ligler F.S., Dickey M.D., Gu Z. (2015). Transformable liquid–metal nanomedicine. Nat. Commun..

[196] Henderson B., Khodabakhsh A., Metsälä M., Ventrillard I., Schmidt F.M., Romanini D., Ritchie G.A.D., te Lintel Hekkert S., Briot R., Risby T. (2018). Laser spectroscopy for breath analysis: Towards clinical implementation. Appl. Phys. B.

[197] Hayes D., Matsukevich D.N., Maunz P., Hucul D., Quraishi Q., Olmschenk S., Campbell W., Mizrahi J., Senko C., Monroe C. (2010). Entanglement of atomic qubits using an optical frequency comb. Phys. Rev. Lett..

[198] Sletten L.R., Moores B.A., Viennot J.J., Lehnert K.W. (2019). Resolving phonon Fock states in a multimode cavity with a double-slit qubit. Phys. Rev. X.

[199] Tabrizian R., Ghatge M. (2018). Phononic Frequency Synthesizer. U.S. Patent.

[200] Ansari A., Park M. (2019). Piezoelectric Resonant-Based Mechanical Frequency Combs. U.S. Patent Application.

